# Minimally Invasive Versus Open Pancreaticoduodenectomy for Distal Cholangiocarcinoma: An Updated Disease-Specific Systematic Review and Meta-Analysis

**DOI:** 10.3390/cancers18091328

**Published:** 2026-04-22

**Authors:** Yi Li, Yulin Lei, Wenli Yang, Wen Zhong, Ran Cui

**Affiliations:** 1 Section for Day Surgery, Department of General Surgery, The Third People’s Hospital of Chengdu, The Affiliated Hospital of Southwest Jiaotong University, No. 19 Yangshi Road, Chengdu 610031, China; lyi1980@126.com; 2 Department of Oncology, The Affiliated Hospital of Southwest Medical University, Luzhou 646000, China; leiyulin1997@163.com; 3 Department of General Medicine, The Third People’s Hospital of Chengdu, Chengdu 610014, China; yangwl0820@163.com; 4 Department of Respiratory and Critical Care Medicine, The First People’s Hospital of Neijiang, Neijiang 641000, China

**Keywords:** distal cholangiocarcinoma, biliary tract cancer, pancreaticoduodenectomy, minimally invasive surgery, laparoscopic pancreaticoduodenectomy, robotic pancreaticoduodenectomy, systematic review, meta-analysis

## Abstract

**Background/Objectives:** Distal cholangiocarcinoma is a rare biliary tract cancer typically treated with pancreaticoduodenectomy. Comparative evidence specifically addressing minimally invasive versus open pancreaticoduodenectomy for this disease remains scarce. **Methods:** We conducted an updated systematic review and pairwise meta-analysis of comparative studies limited to distal cholangiocarcinoma. Binary outcomes were summarized as odds ratios, continuous outcomes as mean differences, and overall survival as hazard ratios. The primary survival analysis included only directly reported hazard ratios from prespecified matched or weighted cohorts; hazard ratios reconstructed from Kaplan–Meier curves were examined only in sensitivity analyses. **Results:** Six retrospective comparative studies involving 1623 patients met the inclusion criteria. Minimally invasive surgery was associated with lower blood loss (mean difference, −104.93 mL; 95% CI, −145.30 to −64.57; I^2^ = 16.3%). No clear differences were found in clinically relevant postoperative pancreatic fistula (odds ratio, 1.03; 95% CI, 0.85 to 1.25), major morbidity (odds ratio, 0.96; 95% CI, 0.64 to 1.43), or R0 resection (odds ratio, 1.22; 95% CI, 0.96 to 1.56). In the primary overall survival analysis based on directly reported hazard ratios, the pooled hazard ratio was 0.93 (95% CI, 0.57 to 1.52; I^2^ = 1.3%). In the sensitivity analysis incorporating eligible reconstructed hazard ratios, the pooled hazard ratio was 0.88 (95% CI, 0.73 to 1.05). In an exploratory recurrence-related survival family analysis based on directly reported estimates, the pooled hazard ratio was 0.95 (95% CI, 0.83 to 1.07; I^2^ = 0.0%). **Conclusions:** Minimally invasive pancreaticoduodenectomy may reduce blood loss without clear evidence of worse major postoperative or oncologic outcomes in distal cholangiocarcinoma. However, the available evidence is entirely observational and should be interpreted with caution.

## Simple Summary

Distal cholangiocarcinoma is an uncommon cancer of the lower bile duct. The standard operation is pancreaticoduodenectomy (the Whipple procedure). Surgeons are increasingly using laparoscopic or robotic approaches for this operation, but it is still unclear whether these less invasive techniques are as safe and effective as open surgery for this specific cancer. In the comparative studies included in this review, minimally invasive surgery was mainly associated with lower blood loss. For other outcomes—such as major complications, complete tumor removal, overall survival, and exploratory recurrence-related survival endpoints—no clear signal of difference from open surgery was observed. Because the available evidence comes only from small retrospective studies, these findings should be interpreted carefully. At present, they support selective use in experienced centers rather than a broad claim that one approach is superior or oncologically equivalent.

## 1. Introduction

Cholangiocarcinoma is a heterogeneous group of biliary tract cancers that vary substantially depending on anatomic site, with crucial implications for surgical therapy, pathologic assessment, and oncologic interpretation [1,2,3,4,5,6,7]. These distinctions are especially consequential in the synthesis of evidence. Distal cholangiocarcinoma should not be assumed to operate like intrahepatic or perihilar disease or to be fully represented by mixed periampullary cohorts alone. Accordingly, current guidelines and surgical reviews continue to regard distal cholangiocarcinoma as a distinct clinical entity, most commonly treated with pancreaticoduodenectomy when resection is feasible [8].

For patients with resectable distal cholangiocarcinoma, pancreaticoduodenectomy remains the sole potentially curative treatment. In this context, the comparison between minimally invasive pancreaticoduodenectomy (MIPD) and open pancreaticoduodenectomy (OPD) extends beyond perioperative safety. It must also address oncologic adequacy, including margin status, lymph-node evaluation, and long-term survival [9,10,11,12]. Comparative studies focusing specifically on distal cholangiocarcinoma have become increasingly available in recent years, and several matched or weighted analyses have directly evaluated MIPD against OPD [13,14,15,16,17,18]. Nevertheless, the evidence base remains limited. All published studies are retrospective, sample sizes are modest, and interpretation is complicated by heterogeneity in minimally invasive platforms, stage selection, pathologic definitions, and the reporting of survival outcomes.

The question has become more relevant as minimally invasive pancreatic surgery has matured. International consensus statements have helped standardize terminology, define implementation principles, and support structured training and adoption, particularly for robotic approaches [19,20,21,22,23,24,25,26,27,28]. Nevertheless, broader uptake does not remove the influence of center volume, learning curve, perioperative care pathways, and case selection, all of which remain major determinants of outcomes after pancreaticoduodenectomy regardless of operative approach [29,30,31,32,33].

Several previous evidence syntheses have examined related aspects of this topic, including the disease-specific pairwise meta-analysis by Domene and colleagues, the individual-patient-data meta-analysis of non-pancreatic periampullary cancers by Uijterwijk and colleagues [34], and the cholangiocarcinoma-focused network meta-analysis by Dong and colleagues [35]. However, these prior syntheses either predated the recent Gao 2025 cohort [18], combined distal cholangiocarcinoma with broader periampullary or cholangiocarcinoma populations, or did not clearly distinguish directly reported hazard ratios from estimates reconstructed from Kaplan–Meier curves. This is especially salient in a small observational evidence base, because apparent precision can easily be achieved while additional layers of assumption are introduced. Distal cholangiocarcinoma also has practical oncologic features that warrant disease-specific interpretation, particularly with respect to margin assessment and lymph-node evaluation after pancreaticoduodenectomy. Even when the operative procedure is nominally the same, differences in specimen handling, reporting standards, and pathology workflow may shift pooled pathologic outcomes in ways not attributable to surgical approach alone.

For these reasons, we conducted an updated, disease-specific systematic review and pairwise meta-analysis comparing MIPD versus OPD for distal cholangiocarcinoma. In addition to incorporating the recently published Gao 2025 cohort [18], we prespecified a conservative survival evidence hierarchy that uses directly reported hazard ratios from matched or weighted comparative analyses as the primary evidence while reserving Kaplan–Meier reconstruction for sensitivity analysis. We also performed a structured audit for potential cohort overlap before pooling. Our intent was to produce an updated estimate that remains clinically interpretable and appropriately cautious for a rare disease where the available comparative studies are retrospective and heterogeneous.

## 2. Materials and Methods

### 2.1. Study Design and Reporting

This updated disease-specific systematic review and pairwise meta-analysis compared MIPD with OPD for distal cholangiocarcinoma and was reported in accordance with the PRISMA 2020 statement [36]. This review was registered in PROSPERO (CRD420261344758).

### 2.2. Search Strategy and Study Selection

PubMed, Embase, Web of Science Core Collection, the Cochrane Library, and Google Scholar were searched from database inception to 21 March 2026 using controlled vocabulary (where available) and free-text terms for distal cholangiocarcinoma, pancreaticoduodenectomy or Whipple procedure, and minimally invasive, laparoscopic, or robotic surgery. Searches were restricted to English-language records to facilitate consistent screening and extraction of disease-specific comparative data, particularly adjustment details and mature survival reporting. We acknowledge that this restriction introduces the potential for language bias. Gray literature sources and conference abstracts were not systematically searched and were not eligible for primary quantitative pooling. Reference lists of included studies and relevant reviews were hand-searched for additional eligible reports. Hand-searching was limited to identifying additional peer-reviewed full-text reports meeting the eligibility criteria. The database-specific search strategies are provided in the Supplementary Materials. Two reviewers independently screened titles and abstracts, followed by full-text review for eligibility. Disagreements were resolved by discussion and, when necessary, consultation with a third reviewer.

Search results from each database were exported and combined in a reference-management workflow prior to screening. Duplicates were removed before title and abstract screening. Screening was performed in two stages, with independent full-text assessment for potentially eligible records. Reasons for exclusion at the full-text stage were documented at the time of screening to ensure transparency and to support PRISMA reporting (Table S1).

### 2.3. Eligibility Criteria

Eligible studies were comparative human studies evaluating pancreaticoduodenectomy for pathologically confirmed distal cholangiocarcinoma, with one MIPD arm and one OPD arm. Retrospective comparative cohorts using propensity-score matching, inverse probability of treatment weighting, or comparable adjustment strategies were eligible. Conference abstracts, non-peer-reviewed reports, and other gray literature were excluded from primary pooling due to the inherently limited availability of disease-specific extractable data, adjustment details, and mature survival information in these formats. Non-comparative case series, technical reports, benign-disease cohorts, and mixed periampullary studies without extractable disease-specific distal cholangiocarcinoma data were also excluded. The main clinical comparison was MIPD versus OPD; therefore, laparoscopic, robotic, and mixed minimally invasive cohorts were eligible for the primary pairwise synthesis, whereas platform-specific interpretation was reserved for contextual discussion.

### 2.4. Data Extraction and Risk of Bias

Two reviewers independently extracted the study characteristics, perioperative outcomes, pathologic end points, and survival estimates using a standardized data-extraction form (Tables S2–S8). Risk of bias was assessed with the Newcastle-Ottawa Scale because all included studies were retrospective comparative cohorts [37]. To contextualize the strength of inference for clinically emphasized endpoints, we additionally summarized the certainty of evidence using a GRADE-based framework at the outcome level, considering risk of bias, inconsistency, indirectness, imprecision, and potential reporting bias. Because all included studies were observational and several outcomes were limited by small sample size and wide confidence intervals, the GRADE summary was used to support cautious interpretation rather than to generate definitive practice recommendations. The detailed GRADE assessment is provided in the Supplementary Materials (Table S9).

### 2.5. Outcomes and Study-Level Interpretability Considerations

The clinically emphasized outcomes included overall survival (OS), R0 resection, lymph-node yield, clinically relevant postoperative pancreatic fistula (CR-POPF), and major morbidity. Blood loss, operative time, length of stay, delayed gastric emptying, and early mortality were treated as secondary perioperative outcomes. Textbook outcome was retained descriptively because it was available in only one included study [18].

Several study-level considerations were specified before extraction (Table S10). Lee 2023 was considered stage restricted because only stage I–IIb distal cholangiocarcinoma was included [16]. Uijterwijk 2023 defined R1 disease using a 1 mm rule, so its margin data were interpreted separately from studies using less strict definitions [11,17]. Kim 2022 and Uijterwijk 2023 combined laparoscopic and robotic procedures within a single minimally invasive group, which limited platform-specific interpretation [13,17]. Xu 2022 and Gao 2025 were checked for possible duplicate reporting [15,18]. The explicit adjudication rule incorporated recruitment window, center identity, author overlap, and comparison family. While a strict quantitative threshold (e.g., excluding studies with ≥2 shared authors) might seem theoretically desirable, such rigid criteria are empirically unreliable for multicenter retrospective databases where institutional identities are deliberately anonymized, and collaborative author lists dynamically change across publication years. Relying on author counts alone risks erroneously excluding independent datasets or, conversely, failing to detect true overlap between distinct research groups using the same national registry. Therefore, we avoided arbitrary quantitative thresholds and instead adopted a comprehensive qualitative adjudication matrix. Because their recruitment windows only partly overlapped, there was limited author overlap, and no confirmed center-level duplication was identified; the participating center sets were judged to be clinically distinct (Table S11).

### 2.6. Statistical Analysis and Survival Sensitivity Strategy

Binary outcomes were pooled as odds ratios, continuous outcomes as mean differences, and survival outcomes as hazard ratios using random-effects models with REML estimation and Hartung–Knapp small-sample adjustment, in line with contemporary guidance [38]. When studies reported continuous outcomes as medians with ranges or interquartile ranges, we applied validated estimation methods to approximate sample means and standard deviations [39,40].

We attempted to minimize avoidable analytic assumptions. In particular, we did not impute dispersion measures when they were not reported and could not be derived from available summary statistics, and we did not treat non-identical endpoints as interchangeable in the primary analysis. For outcomes known to be sensitive to local practice patterns, such as length of stay and operative time, we interpreted heterogeneity clinically rather than assuming that a single pooled estimate represented a stable “true” effect across settings.

Heterogeneity was described with the I^2^ statistic and interpreted clinically rather than mechanically. Small-study effect testing and formal assessment of funnel plot asymmetry were not performed because no pooled endpoint included ten or more studies; in line with methodological guidance, such testing was considered underpowered and potentially misleading. All quantitative analyses were performed in R v4.5.1 (R Foundation for Statistical Computing, Vienna, Austria), primarily using the meta package (v8.2-1) and metafor package (v4.8-0); survival analyses related to reconstructed individual patient data used the survival package (v3.8-6). For arm-level binary and continuous outcomes, actual matched cohorts were strictly preferred. When matching was not available but raw arm-level counts were, crude actual-cohort data were preferred for perioperative pooling over weighted pseudo-cohorts, whereas their inverse probability of treatment weighting (IPTW) total-effect hazard ratios were preferred for survival.

Two investigators independently digitized each eligible Kaplan–Meier panel and checked arm assignment, axis calibration, and pooling eligibility, with disagreements resolved by consensus (Table S12).

The primary overall-survival synthesis was restricted to directly reported hazard ratios from the preferred matched or weighted comparative analyses. Due to the inconsistent endpoint definitions reported across the included studies (including disease-free survival, recurrence-free survival, and disease-free interval), a standardized pooled analysis of recurrence-free survival was not feasible. Therefore, we pre-specified an exploratory recurrence-related survival family analysis combining these directly reported estimates, explicitly acknowledging the limitations of this approach. Directly reported hazard ratios from eligible matched or weighted comparative analyses were pooled for the primary overall-survival synthesis. Studies providing matched-cohort Kaplan–Meier curves without directly reported hazard ratios were reserved for sensitivity analysis to avoid mixing direct and reconstructed estimates in the main model. Specifically, these reconstructed estimates were incorporated into a combined direct-plus-reconstructed sensitivity model. The hierarchical workflow for survival data processing and the evidence-level selection criteria is visually summarized in Figure S1. We deliberately chose not to present a standalone ‘Kaplan–Meier-only’ pooled model. Given that only two matched cohorts yielded eligible reconstructed survival data, a standalone reconstructed model would be statistically underpowered, highly sensitive to digitization assumptions, and prone to overinterpretation. Retaining a combined sensitivity model appropriately balances the need for comprehensive data inclusion with the methodological requirement for valid, cautious inference.

## 3. Results

### 3.1. Study Selection and Study Characteristics

**Figure 1:**
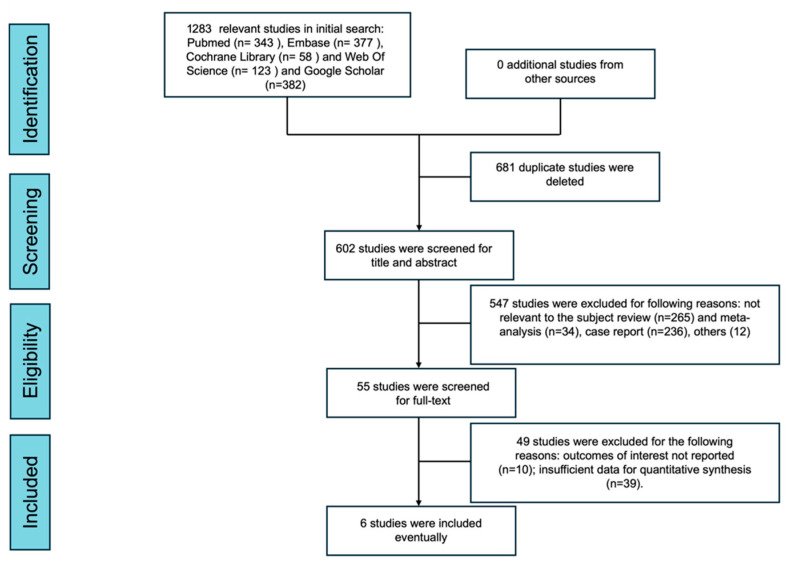
PRISMA 2020 flow diagram of study identification, screening, eligibility assessment, and inclusion.

After database searching, deduplication, and two-stage screening, six comparative studies met the disease-specific eligibility criteria for quantitative synthesis (Figure 1) [13,14,15,16,17,18]. Across the analytic datasets retained for pooling, up to 1623 patients contributed data. Most included studies were conducted in East Asia, with one international multicenter cohort. Propensity-score matching or weighting was used in most studies. Table 1 summarizes the included cohorts and their main interpretability caveats.

Manual screening for possible duplicate reporting found no confirmed center-level duplication between Xu 2022 [15] and Gao 2025 [18]; the participating center sets were judged to be different, and both studies were retained in the main analyses. Newcastle-Ottawa Scale performance is summarized in Figure S2 and Table S13. Overlap-sensitive reruns excluding each potentially overlapping cohort in turn are summarized in Figure S3.

### 3.2. Perioperative Outcomes

**Figure 2:**
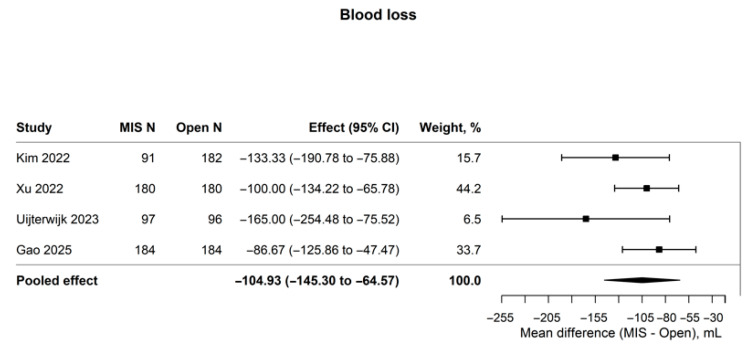
Forest plot of intraoperative blood loss after MIPD versus OPD for distal cholangiocarcinoma. Author–year labels in the figure correspond to references [13,15,17,18].

**Figure 3:**
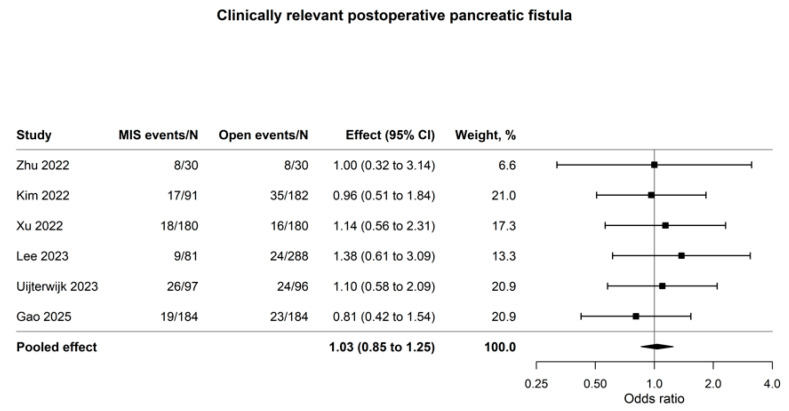
Forest plot of clinically relevant postoperative pancreatic fistula after MIPD versus OPD for distal cholangiocarcinoma. Author–year labels in the figure correspond to references [13,14,15,16,17,18].

The most consistent perioperative signal favoring MIPD was lower blood loss (four studies; n = 1194; mean difference, −104.93 mL; 95% CI, −145.30 to −64.57; I^2^ = 16.3%; Figure 2). A mean reduction of approximately 105 mL, while modest in absolute terms, may translate to clinically meaningful reductions in intraoperative transfusion requirements in selected patients. In contrast, clinically relevant postoperative pancreatic fistula, major morbidity, delayed gastric emptying, and early mortality showed no clear between-group differences, with effect estimates clustered around the null and little statistical heterogeneity (Table 2; Figure 3 and Figures S4–S6).

Operative time and postoperative length of stay were directionally unstable across studies and showed substantial heterogeneity. MIPD tended to be associated with longer operative time and shorter hospitalization, but neither outcome was considered a robust cross-study effect (Table 2; Figures S7 and S8).

### 3.3. Pathologic and Oncologic Adequacy

**Figure 4:**
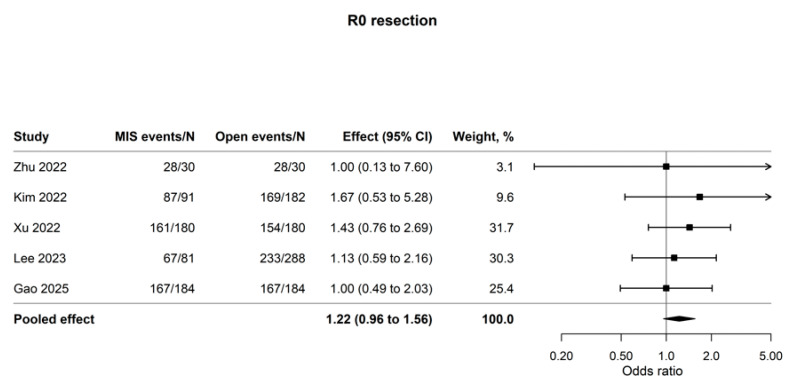
Forest plot of R0 resection after MIPD versus OPD for distal cholangiocarcinoma. Author–year labels in the figure correspond to references [13,14,15,16,18].

Five studies comprising 1430 patients contributed to the primary R0 analysis [13,16,18]. Uijterwijk 2023 was excluded from this pooled estimate because it defined R1 using a strict tumor-within-1-mm rule, which was considered insufficiently comparable with the margin definitions used in the other cohorts (Figure S9) [17]. In this harmonized five-study subset, the pooled estimate numerically favored MIPD but remained statistically inconclusive (OR, 1.22; 95% CI, 0.96 to 1.56; I^2^ = 0.0%; Figure 4). Notably, in a targeted sensitivity analysis replacing the crude counts of Lee 2023 with their inverse probability of treatment weighted (IPTW) pseudo-cohort values, the pooled estimate for R0 resection reached nominal statistical significance favoring MIPD (OR, 1.25; 95% CI, 1.04 to 1.52; Table S14) [16]. However, because these weighted pseudo-cohort counts are mathematically derived rather than representing actual matched patients, this structure-sensitive finding requires cautious interpretation.

Lymph-node yield, available from five studies and 1563 patients, showed no clear difference (mean difference, −0.90; 95% CI, −4.41 to 2.61; I^2^ = 88.2%; Figure S10). These pooled data do not show an obvious compromise of core pathologic adequacy, but interpretation still requires caution because pathology handling differed across studies, minimally invasive platforms were sometimes grouped together, and margin definitions were not fully uniform. To explore potential clinical heterogeneity and address differences in surgical platforms and geographical settings, a descriptive subgroup summary is provided in Table S4.

### 3.4. Overall Survival and Sensitivity Analyses

**Figure 5:**
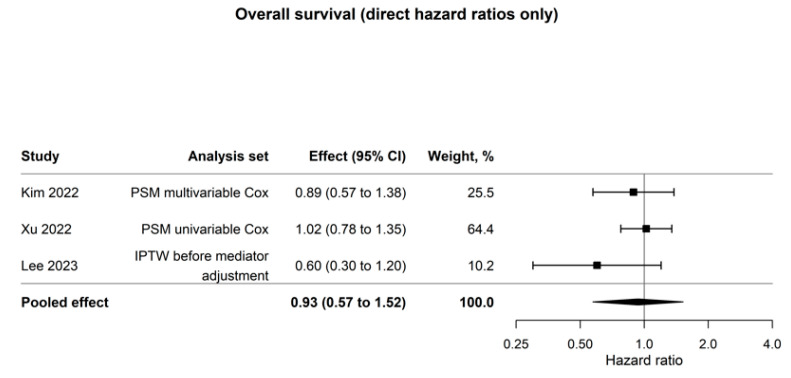
Forest plot of overall survival after MIPD versus OPD for distal cholangiocarcinoma using directly reported hazard ratios only. Author–year labels in the figure correspond to references [13,15,16].

The primary overall-survival synthesis included only directly reported hazard ratios from the preferred matched or weighted comparative analyses. Three studies and 1002 patients contributed to this model, yielding a pooled hazard ratio of 0.93 (95% CI, 0.57 to 1.52; I^2^ = 1.3%; Figure 5), with no clear survival difference between MIPD and OPD [13,15,16]. Zhu 2022 [14], the matched cohort in Uijterwijk 2023, and Gao 2025 mainly provided matched-cohort Kaplan–Meier survival data and were therefore reserved for sensitivity analysis rather than the primary estimate [17,18]. This separation avoided combining directly reported hazard ratios with reconstructed estimates in the main model.

Under the prespecified Kaplan–Meier reconstruction eligibility rule, reconstructed matched-cohort hazard ratios from Gao 2025 [18] and Uijterwijk 2023 [17] were eligible for pooling. Data from Zhu 2022 [14] could not be pooled because its matched-cohort Kaplan–Meier panel lacked a usable numbers-at-risk table to support reliable reconstruction. Adding the two eligible reconstructed estimates to the three-study direct-HR set yielded a five-study sensitivity model comprising 1563 patients, with a pooled hazard ratio of 0.88 (95% CI, 0.73 to 1.05; I^2^ = 0.0%; Figure S11), consistent in direction with the primary analysis. For completeness, an exploratory pooling of the two reconstructed matched-cohort estimates alone (Gao 2025 [18] and Uijterwijk 2023 [17]) yielded a directionally similar but statistically underpowered result (HR 0.84, 95% CI 0.65 to 1.08) and was therefore not used for primary inference.

In the exploratory recurrence-related survival family analysis, combining directly reported disease-free or recurrence-free survival estimates, three studies (1002 patients) yielded a pooled hazard ratio of 0.95 (95% CI, 0.83 to 1.07; I^2^ = 0.0%). A broader sensitivity model incorporating the overall-cohort disease-free interval estimate from Uijterwijk 2023 [17] remained directionally similar (HR, 0.93; 95% CI, 0.84 to 1.02; I^2^ = 0.0%).

## 4. Discussion

In this updated disease-specific review, the clearest short-term advantage associated with MIPD was lower blood loss, whereas no clear differences were identified for the major postoperative and oncologic outcomes assessed. Given the observational evidence base and the low certainty of several key endpoints, these findings support the feasibility of selecting patients treated at experienced centers rather than broad claims of superiority or oncologic equivalence.

This update should be interpreted in the context of earlier evidence syntheses. Unlike prior reviews that either predated the Gao 2025 cohort, combined distal cholangiocarcinoma with broader periampullary or cholangiocarcinoma populations, or did not clearly distinguish directly reported from reconstructed survival estimates, the present analysis remained strictly disease-specific and methodologically conservative [34,35,41,42]. In a rare malignancy with a small retrospective literature, that design option prioritizes interpretability over apparent precision.

From an oncologic perspective, overall survival warrants the greatest interpretive emphasis in this review, as the clinical question in distal cholangiocarcinoma extends beyond perioperative safety to the adequacy and durability of cancer control. In the primary model, which was restricted to directly reported hazard ratios derived from matched or weighted comparative analyses, three studies comprising 1002 patients yielded a pooled HR for overall survival of 0.93 (95% CI, 0.57–1.52; I^2^ = 1.3%) [13,15,16]. Although this estimate does not indicate inferior survival after MIPD, the confidence interval remains sufficiently broad to encompass a clinically meaningful benefit or detriment. This imprecision has consequences since the lack of a statistically significant difference should not be regarded as proof of similar oncologic effectiveness. When the two eligible matched-cohort Kaplan–Meier reconstructions from Gao 2025 [18] and Uijterwijk 2023 [17] were incorporated into the prespecified sensitivity model, the pooled HR shifted modestly in favor of MIPD, and the confidence interval narrowed to 0.88 (95% CI, 0.73–1.05; I^2^ = 0.0%). For completeness, separate pooling of the two reconstructed cohorts yielded a directionally consistent estimate (HR 0.84, 95% CI, 0.65–1.08), although this analysis was statistically underpowered and was therefore not used for primary inference [17,18]. The small size of the primary OS model reflects not only the rarity of the disease, but also incomplete survival reporting in matched-cohort studies within the source literature. For example, Zhu 2022 [14] could not be reconstructed reliably because the matched Kaplan–Meier panel did not include an arm-specific numbers-at-risk table [14]. Taken together, these analyses indicate that the absence of an adverse survival signal is not attributable to any single study or modeling approach. At the same time, the apparent improvement in precision in the combined model is due in part to the incorporation of reconstructed rather than directly reported estimates, and that model is therefore more corroborative than definitive.

That caution is further supported by the pathologic findings: although the harmonized primary analysis of R0 resection remained statistically inconclusive, the estimate reached nominal significance when Lee 2023 was analyzed using IPTW-derived pseudo-cohort counts, highlighting the sensitivity of margin outcomes to changes in analytic specification [16]. The exploratory recurrence-related analyses point in the same direction, but they provide corroboration rather than definitive resolution. Pooling the directly reported DFS or RFS estimates from the matched or weighted cohorts yielded an HR of 0.95 (95% CI, 0.83–1.07; I^2^ = 0.0%). A broader sensitivity model, which additionally incorporated the overall-cohort disease-free interval estimate from Uijterwijk 2023, produced a similar estimate (HR 0.93; 95% CI, 0.84–1.02) [17]. These findings are reassuring in that they do not suggest an obvious recurrence disadvantage with MIPD, yet they cannot be regarded as a fully harmonized recurrence endpoint because DFS, RFS, and DFI are related but not interchangeable constructs. This distinction is especially relevant in distal cholangiocarcinoma, where longitudinal ductal spread, periductal infiltration, assessment of the retropancreatic margin, and pathology handling all influence oncologic interpretation [1,9,10,11,17]. The survival signal should therefore be interpreted alongside the similarly non-definitive pathologic findings—R0 resection OR 1.22 (95% CI, 0.96–1.56) and lymph-node yield MD −0.90 (95% CI, −4.41 to 2.61)—rather than considered in isolation [13,16,18]. Put differently, the currently available comparative data suggest the absence of an obvious oncologic disadvantage in selected patients, rather than proving oncologic equivalence. This distinction accords with the very low GRADE certainty assigned to overall survival and R0 resection due to imprecision and potential reporting bias.

Lymph-node yield should be considered independently of these perioperative process measures. In the present review, it was regarded as a clinically salient pathologic outcome rather than a secondary perioperative variable, and its marked heterogeneity (MD, −0.90 nodes; 95% CI, −4.41 to 2.61; I^2^ = 88.2%) likely reflects variation in both nodal dissection and specimen processing. The study-level pattern was likewise inconsistent rather than uniformly unfavorable to MIPD: Kim 2022 [13] reported a lower nodal count in the minimally invasive arm, whereas Xu 2022 [15], Uijterwijk 2023 [17], and Gao 2025 [18] reported neutral or slightly favorable results for MIPD. This pattern is more consistent with differences in pathology workflow and nodal counting practices than with a reproducible access-related oncologic deficit.

Reduced blood loss was the most reproducible short-term perioperative finding favoring MIPD (MD, −104.93 mL; 95% CI, −145.30 to −64.57), whereas the effect estimates for CR-POPF (OR, 1.03; 95% CI, 0.85–1.25) and major morbidity (OR, 0.96; 95% CI, 0.64–1.43) remained close to the null. This relative consistency suggests that the observed reduction in blood loss reflects a genuine signal of intraoperative efficiency rather than a study-specific artifact. From a clinical perspective, this pattern is plausible. During pancreaticoduodenectomy, blood loss is particularly responsive to operative exposure, magnified visualization, and control of small venous tributaries, advantages that may become evident relatively early as minimally invasive programs mature [21,22,23,24,25,26,27]. In contrast, severe postoperative events after resection for distal cholangiocarcinoma are shaped by a broader range of gland-, disease-, and system-level factors, including pancreatic texture, duct caliber, biliary instrumentation or contamination, the need for vascular resection, reconstructive strategy, and the center’s capacity to recognize and rescue complications [29,30,31,32,33]. The approximately 105-mL reduction in blood loss should therefore be interpreted as an indicator of procedural efficiency in selected cases, rather than as a surrogate for lower biologic fistula risk or an assured reduction in severe morbidity. Because the included cohorts did not report reconstruction and pancreatic anastomotic techniques with sufficient granularity and standardization, the present data cannot determine whether differences in reconstruction modified the CR-POPF signal [21,22,23,24,25,26,27,43].

Other process-sensitive perioperative outcomes were markedly less consistent across studies. Operative time tended to be longer with MIPD (MD, 52.96 min; 95% CI, −10.76 to 116.69; I^2^ = 95.7%), whereas length of stay appeared shorter (MD, −2.27 days; 95% CI, −5.22 to 0.68; I^2^ = 82.6%), but neither estimate was sufficiently consistent to support a uniform effect across studies. This heterogeneity is clinically explicable. In pancreaticoduodenectomy, operative time reflects not only the access route but also the learning curve stage, setup demands, and trainee participation. Notably, operative duration was nearly identical between approaches in the post-learning-curve Gao cohort [18] and differed only modestly in the multicenter robotic Xu cohort [15]. This suggests that any time penalty is program-dependent rather than intrinsic to the minimally invasive approach itself. Future integration of artificial intelligence into robotic workflows [44] may eventually standardize these process-sensitive outcomes. Length of stay is even more dependent on institutional processes, varying with the implementation of enhanced recovery measures, drain management, policies for diet advancement, discharge thresholds, and the availability of post-discharge monitoring or interventional rescue pathways [29,30,31,32,33]. These endpoints therefore characterize how a center delivers pancreaticoduodenectomy as much as they reflect the operative approach itself, and they should not be interpreted as transferable efficiency metrics.

This review is strengthened by its disease-specific scope and by the cautious interpretation of survival outcomes. Even so, the available evidence remains limited. All included studies were retrospective comparative cohort studies, only six were suitable for quantitative synthesis, and nonrandom selection into MIPD likely persisted despite the use of matching or weighting. The available data were also concentrated in high-volume East Asian centers, which may limit the generalizability of the findings to other practice settings. At the review level, limiting eligibility to English-language, peer-reviewed reports and excluding gray literature may have introduced language and publication bias, although these decisions were intended to preserve the extractability and comparability of disease-specific data. Additional uncertainty arose because laparoscopic and robotic procedures were frequently grouped within a single minimally invasive category, margin definitions were not fully standardized across studies, and survival reporting for matched cohorts remained incomplete in several datasets. Several key estimates were likewise imprecise, with confidence intervals sufficiently wide to encompass potentially meaningful differences in either direction. These constraints are reflected in the GRADE assessment, which rated evidence for blood loss as low certainty and evidence for CR-POPF, major morbidity, R0 resection, and overall survival as very low certainty. The present findings should therefore be interpreted as indicating feasibility in selected settings rather than as definitive evidence of equivalence.

Future progress will require prospectively collected, multicenter data rather than additional small retrospective comparisons. Disease-specific multicenter datasets should report platform type, conversion according to intention-to-treat principles, reconstructive technique, margin definitions, node-processing protocols, thromboembolic events, definitions of early mortality, and directly reported hazard ratios accompanied by usable numbers-at-risk tables. Technologies for intraoperative margin assessment may enhance real-time decision-making during minimally invasive resection, but they should not be conflated with final pathological assessment. AI-assisted workflow support may eventually become relevant to technology-enabled surgery, although such developments remain outside the comparative evidence base reviewed here.

## 5. Conclusions

This review suggests that the principal advantage of MIPD in disease-specific comparative studies is reduced blood loss. Nevertheless, the current evidence base remains limited, selective, and methodologically heterogeneous, and does not permit conclusions regarding oncologic equivalence or overall superiority. MIPD may therefore be considered a feasible option for carefully selected patients in experienced centers. Prospective multicenter studies with standardized pathology assessment and survival reporting are needed to support more definitive comparative evaluation.

## Figures and Tables

**Table 1:** Characteristics of the included comparative studies evaluating MIPD versus OPD for distal cholangiocarcinoma.

Study	Country/Setting	Design and Adjustment	Analytic Cohort	Comparison
Zhu et al., 2022 [14]	China; single center	Retrospective comparative cohort; 1:1 PSM	Matched cohort, 30 vs. 30	LPD vs. OPD
Kim et al., 2022 [13]	Korea; 2 tertiary centers	Retrospective comparative cohort; 1:2 PSM	Matched cohort, 91 vs. 182	MIPD vs. OPD
Xu et al., 2022 [15]	China; 5 centers	Retrospective multicenter comparative cohort; 1:1 PSM	Matched cohort, 180 vs. 180	RPD vs. OPD
Lee et al., 2023 [16]	Korea; single tertiary center	Retrospective comparative cohort; IPTW primary analysis with PSM sensitivity	Actual cohort for arm-level pooling, 81 vs. 288	MIPD vs. OPD
Uijterwijk et al., 2023 [17]	International multicenter cohort (8 centers, 5 countries)	Retrospective international comparative cohort; PSM	Matched cohort, 97 vs. 96	MIPD vs. OPD
Gao et al., 2025 [18]	China; single center	Retrospective comparative cohort; 1:1 PSM after learning curve	Matched cohort, 184 vs. 184	LPD vs. OPD

Abbreviations: PSM, propensity-score matching; IPTW, inverse probability of treatment weighting; MIPD, minimally invasive pancreaticoduodenectomy; LPD, laparoscopic pancreaticoduodenectomy; RPD, robotic pancreaticoduodenectomy; OPD, open pancreaticoduodenectomy.

**Table 2:** Quantitative summary of pooled perioperative, pathologic, and survival outcomes.

Outcome	Studies, No.	Participants, No.	Pooled Estimate (95% CI)	Heterogeneity (I^2^)	Measure
Overall survival (direct HR only)	3	1002	0.93 (0.57 to 1.52)	1.3%	HR
R0 resection	5	1430	1.22 (0.96 to 1.56)	0.0%	OR
Lymph node yield	5	1563	−0.90 (−4.41 to 2.61)	88.2%	MD
CR-POPF	6	1623	1.03 (0.85 to 1.25)	0.0%	OR
Major morbidity	5	1563	0.96 (0.64 to 1.43)	0.0%	OR
Blood loss, mL	4	1194	−104.93 (−145.30 to −64.57)	16.3%	MD
Operative time, min	5	1563	52.96 (−10.76 to 116.69)	95.7%	MD
Length of stay, d	5	1563	−2.27 (−5.22 to 0.68)	82.6%	MD
DGE	4	981	0.89 (0.63 to 1.25)	0.0%	OR
Early mortality	4	1194	0.85 (0.43 to 1.68)	0.0%	OR
OS sensitivity (direct HR + reconstructed HR)	5	1563	0.88 (0.73 to 1.05)	0.0%	HR

Abbreviations: OR, odds ratio; MD, mean difference; HR, hazard ratio; CR-POPF, clinically relevant postoperative pancreatic fistula; DGE, delayed gastric emptying; d, days. The direct-plus-reconstructed survival model is sensitivity only.

## Data Availability

All data analyzed in this study are contained within the article and its Supplementary Materials. Extraction sheets, analytic code, and additional review materials are available from the corresponding authors on reasonable request.

## Supplementary Materials

The following supporting information can be downloaded at: https://www.mdpi.com/article/10.3390/cancers18091328/s1, Supplementary Methods: database-adapted search strategies, outcome-harmonization rules, survival modeling framework, and cohort-screening notes; Supplementary Results: overlap-sensitive reruns and Kaplan–Meier reconstruction sensitivity analysis; Table S1 shows the full-text exclusion source audit. Table S2 shows the full study characteristics and extraction-ready cohort details. Table S3 shows the outcome definitions, harmonization rules, and study-specific caveats. Table S4 shows the study-level extraction structure for pooled outcomes. Table S5 shows the descriptive heterogeneity summary: study-level characteristics and directional effects. Table S6 shows the binary outcome input table. Table S7 shows the continuous outcome input table. Table S8 shows the direct survival input table. Table S9 shows the GRADE-based certainty assessment for key outcomes. Table S10 shows the pre-defined interpretability framework. Table S11 shows the cohort-screening overlap audit and adjudication notes. Table S12 shows the Kaplan–Meier digitization and reconstruction log. Table S13 shows the Newcastle-Ottawa Scale scoring. Table S14 shows the leave-one-out and overlap-sensitive rerun summary. Figure S1 shows the survival data evidence hierarchy and reconstruction workflow. Figure S2 shows the Newcastle-Ottawa Scale heatmap. Figure S3 shows the overlap-sensitive reruns for overall survival. Figure S4 shows the major morbidity forest plot. Figure S5 shows the delayed gastric emptying forest plot. Figure S6 shows the early mortality forest plot. Figure S7 shows the operative time forest plot. Figure S8 shows the postoperative length of stay forest plot. Figure S9 shows the alternative R0 sensitivity model including the 1-mm margin-rule cohort. Figure S10 shows the lymph-node yield forest plot. Figure S11 shows the direct plus reconstructed overall-survival sensitivity model.

## Abbreviations

The following abbreviations are used in this manuscript:

APC, article processing charge; CI, confidence interval; CR-POPF, clinically relevant postoperative pancreatic fistula; DGE, delayed gastric emptying; HR, hazard ratio; IPTW, inverse probability of treatment weighting; KM, Kaplan–Meier; MD, mean difference; MIS, minimally invasive surgery; MIPD, minimally invasive pancreaticoduodenectomy; NOS, Newcastle-Ottawa Scale; OPD, open pancreaticoduodenectomy; OR, odds ratio; PSM, propensity-score matching; R0, microscopically margin-negative resection; R1, microscopically margin-positive resection; REML, restricted maximum likelihood.
