# Supplementary material for: Minimally Invasive Versus Open Pancreaticoduodenectomy for Distal Cholangiocarcinoma: An Updated Disease-Specific Systematic Review and Meta-Analysis
Source: Cancers (Basel). 2026 Apr 22;18(9):1328. doi: 10.3390/cancers18091328 (PMC13162705; doi:10.3390/cancers18091328)
Supplement: Supplementary file 1 [file cancers-18-01328-s001.zip › cancers-4254693-supplementary.pdf]

Supplementary Materials

1. Supplementary Methods

1.1. Search Strategy

Electronic searches were performed without publication-period restrictions and were restricted to English-language records. The review question was restricted to distal cholangiocarcinoma or distal bile duct cancer treated by pancreaticoduodenectomy, comparing minimally invasive versus open surgery. The core concept blocks were disease, procedure, and operative approach. Reference lists of included studies and relevant reviews were also screened to identify additional eligible peer-reviewed full-text reports.

Google Scholar was used as a supplementary source for identifying potentially eligible peer-reviewed full-text reports; screening was limited to the first 500 results (or fewer if fewer were returned) sorted by relevance on 21 March 2026.

PubMed

(
("Cholangiocarcinoma"[Mesh] OR "Bile Duct Neoplasms"[Mesh] OR "distal cholangiocarcinoma"[tiab] OR "distal bile duct cancer"[tiab] OR "distal bile duct tumor"[tiab] OR "distal bile duct tumour"[tiab])
AND
("Pancreaticoduodenectomy"[Mesh] OR "pancreaticoduodenectomy"[tiab] OR "pancreatoduodenectomy"[tiab] OR "Whipple procedure"[tiab] OR "Whipple operation"[tiab])
AND
("Minimally Invasive Surgical Procedures"[Mesh] OR "Laparoscopy"[Mesh] OR "Robotic Surgical Procedures"[Mesh] OR "minimally invasive"[tiab] OR "laparoscopic"[tiab] OR "robotic"[tiab] OR "robot-assisted"[tiab] OR "MIPD"[tiab] OR "LPD"[tiab] OR "RPD"[tiab])
)
AND
English[LA]

Embase

(('cholangiocarcinoma'/exp OR 'bile duct tumor'/exp OR 'distal cholangiocarcinoma':ti,ab OR 'distal bile duct cancer':ti,ab OR 'distal bile duct tumor':ti,ab OR 'distal bile duct tumour':ti,ab) AND
('pancreaticoduodenectomy'/exp OR 'pancreaticoduodenectomy':ti,ab OR 'pancreatoduodenectomy':ti,ab OR 'whipple procedure':ti,ab OR 'whipple operation':ti,ab) AND
('minimally invasive surgery'/exp OR 'laparoscopic surgery'/exp OR 'robotic surgery'/exp OR 'minimally invasive':ti,ab OR 'laparoscopic':ti,ab OR 'robotic':ti,ab OR 'robot-assisted':ti,ab OR 'MIPD':ti,ab OR 'LPD':ti,ab OR 'RPD':ti,ab))
[english]/lim

Cochrane Library

(("distal cholangiocarcinoma" OR "distal bile duct cancer" OR "distal bile duct tumor" OR "distal bile duct tumour") AND
("pancreaticoduodenectomy" OR "pancreatoduodenectomy" OR "Whipple procedure" OR "Whipple operation") AND
("minimally invasive" OR "laparoscopic" OR "robotic" OR "robot-assisted" OR "MIPD" OR "LPD" OR "RPD"))

Web of Science

TS=("distal cholangiocarcinoma" OR "distal bile duct cancer" OR "distal bile duct tumor" OR "distal bile duct tumour")
AND
TS=("pancreaticoduodenectomy" OR "pancreatoduodenectomy" OR "Whipple procedure" OR "Whipple operation")
AND
TS=("minimally invasive" OR "laparoscopic" OR "robotic" OR "robot-assisted" OR "MIPD" OR "LPD" OR "RPD")

Google Scholar

("distal cholangiocarcinoma" OR "distal bile duct cancer" OR "distal bile duct tumour" OR "distal bile duct tumor" OR intrapancreatic)

("pancreaticoduodenectomy" OR pancreatoduodenectomy OR Whipple)

(laparoscopic OR robotic OR "minimally invasive" OR "robot-assisted" OR MIPD OR LPD OR RPD)

-review -meta-analysis -systematic -guideline

Google Scholar was used as a supplementary source for identifying potentially eligible peer-reviewed full-text reports; screening was limited to the first 500 results sorted by relevance on 21 March 2026.

1.2. Outcome Harmonization

R0 resection was pooled as reported in the source articles while explicitly acknowledging the 1 mm rule used in Uijterwijk 2023 [2,3]. Major morbidity was treated as a family end point rather than as a strictly identical definition across all studies; when studies reported Clavien-Dindo grade III or higher or equivalent severe complication structures, they were harmonized into a major morbidity category. Lymph-node yield was pooled as a continuous end point but interpreted cautiously because both operative dissection and pathology handling influence the final count. Length of stay and operative time were analyzed as reported after conversion to mean differences when adequate summary statistics were available.

1.3. Survival Evidence Hierarchy

To ensure methodological clarity, survival data were handled according to a strict predefined stepwise decision framework acting as a textual flowchart. In the first step (Primary Model), we assessed whether the study reported a direct hazard ratio from a matched or weighted comparative analysis. If present, the study was included in the Primary OS Model (e.g., Kim 2022, Xu 2022, Lee 2023). If not, we proceeded to the second step (Sensitivity Model), assessing whether the study provided a matched-cohort Kaplan-Meier curve with a usable numbers-at-risk table. If available, the curve was digitized and the hazard ratio reconstructed using the Guyot et al. framework, with the estimate reserved strictly for the Combined Sensitivity Model (e.g., Gao 2025, Uijterwijk 2023). Finally, in the third step (Narrative Synthesis), if the curve lacked a risk table or provided unstable data, the study was retained for narrative discussion only and not pooled quantitatively (e.g., Zhu 2022).

1.4. Overlap Audit

Possible cohort overlap was assessed using country, recruitment period, center descriptors, author overlap, and comparison family. Study pairs were classified as low, possible, or probable concern by

combining these anchors rather than by relying on a single variable. Xu 2022 and Gao 2025 were considered the only pair requiring explicit adjudication. Because overlap could not be proven, both studies were retained in endpoint-specific analyses, and qualitative reruns excluding each study in turn were used to test conclusion robustness.

2. Supplementary Results

2.1. Overlap-Sensitive Reruns

The structured overlap audit classified Xu 2022 versus Gao 2025 as possible concern and all other study pairs as low concern. Excluding either Gao 2025 or Xu 2022 did not reverse the qualitative interpretation of the perioperative, pathologic, or survival findings. For blood loss, the pooled mean difference remained favorable to minimally invasive surgery after exclusion of Gao 2025 and also after exclusion of Xu 2022. For R0 resection, the direction of the pooled estimate remained numerically favorable to minimally invasive surgery under both leave-one-cohort-out reruns. For overall survival sensitivity modeling, exclusion of Gao 2025 yielded a pooled hazard ratio of 0.92 (95% CI, 0.70 to 1.20), whereas exclusion of Xu 2022 yielded 0.82 (95% CI, 0.68 to 0.97); neither rerun suggested worse survival with minimally invasive surgery.

2.2. Kaplan-Meier Reconstruction Sensitivity

Two matched cohorts were reconstructed for the planned sensitivity analysis. Gao 2025 reconstructed to a hazard ratio of 0.81 (95% CI, 0.62 to 1.05), and the matched Uijterwijk 2023 cohort reconstructed to 0.87 (95% CI, 0.57 to 1.31). Zhu 2022 was reviewed for possible reconstruction but was not pooled because the matched-cohort Kaplan-Meier panel lacked a usable risk table, making curve-only reconstruction insufficiently reliable for the planned sensitivity analysis. When the reconstructed hazard ratios were added to the primary directly reported hazard-ratio model, the pooled estimate moved modestly toward minimally invasive surgery but remained statistically inconclusive. For completeness, an exploratory standalone pooling of only the two reconstructed estimates (Gao 2025 and Uijterwijk 2023) yielded a hazard ratio of 0.84 (95% CI, 0.65 to 1.08). However, as prespecified, this standalone "KM-only" model is statistically underpowered and highly sensitive to digitization assumptions, reinforcing our decision to rely on the direct-HR model for primary inference.

2.3. Exploratory Recurrence-Related Survival Analysis

As pre-specified to address the oncologic efficacy of minimally invasive surgery further, an exploratory recurrence-related survival family analysis was performed. Combining directly reported disease-free (Kim 2022) and recurrence-free (Xu 2022, Lee 2023) survival estimates yielded a pooled hazard ratio of 0.95 (95% CI, 0.83 to 1.07; I^2 = 0.0%). A broader sensitivity model that also incorporated the overall-cohort disease-free interval estimate from Uijterwijk 2023 remained directionally similar (HR, 0.93; 95% CI, 0.84 to 1.02; I^2 = 0.0%).

2.4. Sensitivity Analysis of Arm-Level Data Structure

To address potential bias from mixing actual crude cohorts with matched datasets, we performed targeted sensitivity analyses for the Lee 2023 cohort. Excluding Lee 2023 entirely from arm-level pooling confirmed the robustness of our primary findings; the pooled effect estimates for major morbidity and R0 resection remained statistically non-significant, with no change in the direction of the overall conclusions. Secondarily, replacing the crude arm-level counts with the inverse probability of treatment weighted (IPTW) pseudo-cohort values (81 vs 286) for Lee 2023 yielded a directionally similar major morbidity estimate (OR 0.94, 95% CI 0.63 to 1.41). However, in this specific structure-sensitive rerun, the estimate for R0 resection crossed the threshold of nominal statistical significance (OR 1.25, 95% CI 1.04 to 1.52). While this shift suggests that margin outcomes are sensitive to the choice of dataset structure (crude versus weighted counts), we maintain a cautious interpretation. Because pseudo-cohort counts are derived through weighting rather than actual patient-level matching, this statistically significant result in the rerun does not necessarily override the non-significant finding of the primary matched-cohort analysis, but it does emphasize the need for standardized margin reporting in future studies.

3. Supplementary Tables and Figures

Table S1 shows full-text exclusion source audit. Table S2 shows full study characteristics and extraction-ready cohort details. Table S3 shows outcome definitions, harmonization rules, and study-specific caveats. Table S4 shows study-level extraction structure for pooled outcomes. Table S5 shows descriptive heterogeneity summary: study-level characteristics and directional effects. Table S6 shows binary outcome input table. Table S7 shows continuous outcome input table. Table S8 shows direct survival input table. Table S9 shows GRADE-based certainty assessment for key outcomes. Table S10 shows pre-defined interpretability framework. Table S11 shows cohort-screening overlap audit and

adjudication notes. Table S12 shows Kaplan-Meier digitization and reconstruction log. Table S13 shows Newcastle-Ottawa Scale scoring. Table S14 shows leave-one-out and overlap-sensitive rerun summary.

Figure S1 shows survival data evidence hierarchy and reconstruction workflow. Figure S2 shows Newcastle-Ottawa Scale heatmap. Figure S3 shows overlap-sensitive reruns for overall survival. Figure S4 shows major morbidity forest plot. Figure S5 shows delayed gastric emptying forest plot. Figure S6 shows early mortality forest plot. Figure S7 shows operative time forest plot. Figure S8 shows postoperative length of stay forest plot. Figure S9 shows alternative R0 sensitivity model including the 1-mm margin-rule cohort. Figure S10 shows lymph-node yield forest plot. Figure S11 shows direct plus reconstructed overall-survival sensitivity model.

References

1. Page MJ, McKenzie JE, Bossuyt PM, Boutron I, Hoffmann TC, Mulrow CD, Shamseer L, Tetzlaff JM, Akl EA, Brennan SE *et al*: **The PRISMA 2020 statement: an updated guideline for reporting systematic reviews**. *Bmj* 2021, **372**:n71.
2. Uijterwijk BA, Lemmers DHL, Bolm L, Luyer M, Koh YX, Mazzola M, Webber L, Kazemier G, Bannone E, Ramaekers M *et al*: **Long-term Outcomes After Laparoscopic, Robotic, and Open Pancreatoduodenectomy for Distal Cholangiocarcinoma: An International Propensity Score-matched Cohort Study**. *Ann Surg* 2023, **278**(3):e570-e579.
3. Tjaden C, Hinz U, Klaiber U, Heger U, Springfeld C, Goeppert B, Schmidt T, Mehrabi A, Strobel O, Berchtold C *et al*: **Distal Bile Duct Cancer: Radical (R0 > 1 mm) Resection Achieves Favorable Survival**. *Ann Surg* 2023, **277**(1):e112-e118.
4. Guyot P, Ades AE, Ouwens MJ, Welton NJ: **Enhanced secondary analysis of survival data: reconstructing the data from published Kaplan-Meier survival curves**. *BMC Med Res Methodol* 2012, **12**:9.
5. Wan X, Wang W, Liu J, Tong T: **Estimating the sample mean and standard deviation from the sample size, median, range and/or interquartile range**. *BMC Med Res Methodol* 2014, **14**:135.
6. Luo D, Wan X, Liu J, Tong T: **Optimally estimating the sample mean from the sample size, median, mid-range, and/or mid-quartile range**. *Stat Methods Med Res* 2018, **27**(6):1785-1805.

4. Embedded Supplementary Tables

Table S1. Full-text exclusion source audit.

No.	First Author & Year	Article Title	Reason for Exclusion
1	Yoon YS, 2024	Laparoscopic versus open pancreatoduodenectomy for periampullary tumors: a randomized clinical trial.	Non-DCC or mixed periampullary carcinoma without separate DCC data
2	Fall S, 2025	Laparoscopic versus open pancreaticoduodenectomy for pancreatic or periampullary tumors: a multicenter propensity score-matched comparative study.	Non-DCC or mixed periampullary carcinoma without separate DCC data
3	Chapman BC, 2018	Laparoscopic pancreaticoduodenectomy: changing the management of ampullary neoplasms.	Non-DCC or mixed periampullary carcinoma without separate DCC data
4	Sakuma M, 2024	Laparoscopic versus open pancreaticoduodenectomy for ampullary cancer: A retrospective study.	Non-DCC or mixed periampullary carcinoma without separate DCC data
5	Delitto D, 2016	Oncologic and Perioperative Outcomes Following Selective Application of Laparoscopic Pancreaticoduodenectomy for Periampullary Malignancies.	Non-DCC or mixed periampullary carcinoma without separate DCC data
6	Meyyappan T, 2023	Robotic approach mitigates the effect of major complications on survival after pancreaticoduodenectomy for periampullary cancer.	Non-DCC or mixed periampullary carcinoma without separate DCC data
7	Yin SM, 2021	Short-term outcomes after minimally invasive versus open pancreaticoduodenectomy in elderly patients: a propensity score-matched analysis.	Non-DCC or mixed periampullary carcinoma without separate DCC data
8	Dang C, 2021	Comparison of laparoscopic and open pancreaticoduodenectomy for the treatment of nonpancreatic periampullary adenocarcinomas: a propensity score matching analysis.	Non-DCC or mixed periampullary carcinoma without separate DCC data
9	Mazzola M, 2021	Totally laparoscopic versus open pancreaticoduodenectomy: A propensity score matching analysis of short-term outcomes.	Non-DCC or mixed periampullary carcinoma without separate DCC data
10	Han SH, 2020	The Yonsei experience of 104 laparoscopic pancreaticoduodenectomies: a propensity score-matched analysis with open pancreaticoduodenectomy.	Non-DCC or mixed periampullary carcinoma without separate DCC data
11	Bao PQ, 2014	Retrospective comparison of robot-assisted minimally invasive versus open pancreaticoduodenectomy for periampullary neoplasms.	Non-DCC or mixed periampullary carcinoma without separate DCC data
12	Dokmak S, 2015	Laparoscopic pancreaticoduodenectomy should not be routine for resection of periampullary tumors.	Non-DCC or mixed periampullary carcinoma without separate DCC data

13	Shyr BS, 2024	Survival and surgical outcomes of robotic versus open pancreatoduodenectomy for ampullary cancer: A propensity score-matching comparison.	Non-DCC or mixed periampullary carcinoma without separate DCC data
14	Hakeem AR, 2014	A matched-pair analysis of laparoscopic versus open pancreaticoduodenectomy: oncological outcomes using Leeds Pathology Protocol.	Non-DCC or mixed periampullary carcinoma without separate DCC data
15	Song KB, 2015	Matched Case-Control Analysis Comparing Laparoscopic and Open Pylorus-preserving Pancreaticoduodenectomy in Patients With Periampullary Tumors.	Non-DCC or mixed periampullary carcinoma without separate DCC data
16	Shin SH, 2017	Totally laparoscopic or robot-assisted pancreaticoduodenectomy versus open surgery for periampullary neoplasms: separate systematic reviews and meta-analyses.	Non-DCC or mixed periampullary carcinoma without separate DCC data
17	Uijterwijk BA, 2025	Oncological resection and perioperative outcomes of robotic, laparoscopic and open pancreatoduodenectomy for ampullary adenocarcinoma: a propensity score matched international multicenter cohort study.	Non-DCC or mixed periampullary carcinoma without separate DCC data
18	Uijterwijk BA, 2026	Outcome of Minimally Invasive and Open Pancreatoduodenectomy in Patients With Intestinal and Pancreatobiliary Subtype Ampullary Cancer: An International Multicenter Cohort Study.	Non-DCC or mixed periampullary carcinoma without separate DCC data
19	Menso JE, 2025	Oncological outcome after robot-assisted versus open pancreatoduodenectomy for upfront resectable cancer in the pancreatic head: a nationwide cohort analysis.	Non-DCC or mixed periampullary carcinoma without separate DCC data
20	Zhang J, 2025	Minimally invasive versus open pancreaticoduodenectomy for periampullary tumors: a systematic review and meta-analysis of randomized controlled trials.	Non-DCC or mixed periampullary carcinoma without separate DCC data
21	Podda M, 2020	Robotic-assisted versus open pancreaticoduodenectomy for patients with benign and malignant periampullary disease: a systematic review and meta-analysis of short-term outcomes.	Non-DCC or mixed periampullary carcinoma without separate DCC data
22	Chen K, 2018	Expanding laparoscopic pancreaticoduodenectomy to pancreatic-head and periampullary malignancy: major findings based on systematic review and meta-analysis.	Non-DCC or mixed periampullary carcinoma without separate DCC data
23	Domene S, 2024	Comparative Outcomes of Minimally Invasive Versus Open Pancreatoduodenectomy in Distal Cholangiocarcinoma: A Systematic Review and Meta-Analysis.	Non-DCC or mixed periampullary carcinoma without separate DCC data

24	Kossenas K, 2025	Comparing the operative, oncological, post-operative outcomes and complications of robotic and laparoscopic pancreaticoduodenectomy for the treatment of pancreatic and periampullary cancers: a systematic review and meta-analysis with subgroup analysis.	Non-DCC or mixed periampullary carcinoma without separate DCC data
25	Liu R, 2017	The surgical outcomes of robot-assisted laparoscopic pancreaticoduodenectomy versus laparoscopic pancreaticoduodenectomy for periampullary neoplasms: a comparative study of a single center.	Non-DCC or mixed periampullary carcinoma without separate DCC data
26	Uijterwijk BA, 2023	The clinical implication of minimally invasive versus open pancreatoduodenectomy for non-pancreatic periampullary cancer: a systematic review and individual patient data meta-analysis.	Non-DCC or mixed periampullary carcinoma without separate DCC data
27	Xu S, 2025	Laparoscopic versus robotic pancreaticoduodenectomy for distal cholangiocarcinoma after learning curves of surgeons: a multicenter propensity score-matched study.	Non-DCC or mixed periampullary carcinoma without separate DCC data
28	Palen A, 2025	R1 Endoscopic papillectomy for adenocarcinoma: is complementary pancreatoduodenectomy unavoidable?	Non-DCC or mixed periampullary carcinoma without separate DCC data
29	Chen Y, 2025	Comparison of endoscopic papillectomy and pancreatoduodenectomy for high-grade intraepithelial neoplasia of the duodenal papilla: a retrospective cohort study.	Non-DCC or mixed periampullary carcinoma without separate DCC data
30	Cho A, 2014	Performing simple and safe dunking pancreaticojejunostomy using mattress sutures in pure laparoscopic pancreaticoduodenectomy.	Non-DCC or mixed periampullary carcinoma without separate DCC data
31	Meng LW, 2018	Comparison of Laparoscopic and Open Pancreaticoduodenectomy for the Treatment of Nonpancreatic Periampullary Adenocarcinomas.	Non-DCC or mixed periampullary carcinoma without separate DCC data
32	Langan RC, 2014	Laparoscopic-assisted versus open pancreaticoduodenectomy: early favorable physical quality-of-life measures.	No relevant outcomes reported
33	Wakabayashi T, 2025	Reduced pancreatic fistula rates and comprehensive cost analysis of robotic versus open pancreaticoduodenectomy.	No relevant outcomes reported
34	Chuang SH, 2022	Single-Incision Laparoscopic Pancreatoduodenectomy (Whipple Procedure) - a Pioneer Technical Report.	No relevant outcomes reported
35	Chong EH, 2019	Hybrid Laparoscopic and Robotic Hepatopancreaticoduodenectomy for Cholangiocarcinoma.	No relevant outcomes reported
36	Koh FH, 2017	Minimally Invasive Whipple's Technique for Laparoscopic-Assisted Pylorus-Preserving Pancreaticoduodenectomy.	No relevant outcomes reported

37	Katsumata K, 2025	Robotic Approach to Oncologic Pancreatoduodenectomy in a Case with an Aberrant Hepatic Artery Originating from the Superior Mesenteric Artery.	No relevant outcomes reported
38	Takagi K, 2024	Robotic Pancreatoduodenectomy in Portal Annular Pancreas Using a Hanging Maneuver with Indocyanine Green Fluorescence Imaging.	No relevant outcomes reported
39	Wei Q, 2019	Repair of the portal vein using a hepatic ligamentum teres patch for laparoscopic pancreatoduodenectomy: A case report.	No relevant outcomes reported
40	Huang J, 2024	Laparoscopic Resection of the Middle Bile Duct for Cholangiocarcinoma (with Video).	No relevant outcomes reported
41	Surjan RCT, 2024	ROBOTIC PANCREATICODUODENECTOMY FOR THE TREATMENT OF A MIXED NEUROENDOCRINE-NON-NEUROENDOCRINE NEOPLASM (MINEN) OF THE AMPULLA OF VATER.	No relevant outcomes reported
42	Wang GY, 2026	Robotic Partial Hepatectomy Combined with Pancreaticoduodenectomy for Simultaneous Gallbladder and Common Bile Duct Tumors.	No relevant outcomes reported
43	Jeon SM, 2025	Oncologic effect of preoperative endoscopic sphincterotomy in patients undergoing pancreaticoduodenectomy for ampulla of vater cancer.	No relevant outcomes reported
44	Li D, 2023	Perioperative Outcomes and Long-Term Survival of Laparoscopic Pancreaticoduodenectomy: A Retrospective Study of 653 Cases in a Single Institution.	No relevant outcomes reported
45	Zhang L, 2020	Natural orifice specimen extraction surgery in laparoscopic pancreaticoduodenectomy: A single-center case series.	No relevant outcomes reported
46	Hu XS, 2024	"Five steps four quadrants" modularized en bloc dissection technique for accessing hepatic hilum lymph nodes in laparoscopic pancreaticoduodenectomy.	No relevant outcomes reported
47	Cai X, 2008	Completed laparoscopic pancreaticoduodenectomy.	Insufficient data for quantitative synthesis
48	Fan Y, 2016	Successful Experience of Laparoscopic Pancreaticoduodenectomy and Digestive Tract Reconstruction With Minimized Complications Rate by 14 Case Reports.	Insufficient data for quantitative synthesis
49	Zhang MZ, 2014	Resection of a cholangiocarcinoma via laparoscopic hepatopancreato-duodenectomy: a case report.	Insufficient data for quantitative synthesis

This audit lists all records that reached full-text assessment from database searches and reference-list hand-searching/Google Scholar screening; conference abstracts and non-peer-reviewed items were not advanced to quantitative pooling and were generally not screened at full text unless a peer-reviewed full report was available.

Table S2. Full study characteristics and extraction-ready cohort details.

citation_short	centers	multicenter	design	population	disease_term	stage_restricted	comparison	primary_mis_subgroup	perioperative_dataset_used	survival_dataset_used	adjustment_method	notes
Zhu et al., 2022	China; single centre	No	Retrospective comparative cohort	Pathology-confirmed distal cholangiocarcinoma (DCC); radical PD	dCCA	No	LPD vs OPD	LPD	PSM 30 vs 30	Matched KM for OS; direct HR only from overall cohort Cox model	1:1 PSM	Single-centre DCC cohort; continuous perioperative outcomes reported without dispersion, so several continuous rows are not directly poolable.
Kim et al., 2022	Korea; 2 tertiary centres	Yes	Retrospective comparative cohort	Pathology-confirmed distal cholangiocarcinoma; upfront PD	dCCA	No	MIPD vs OPD	Mixed-MIPD	PSM 91 vs 182	PSM multivariable HRs (DFS/OS)	1:2 PSM	Most complete matched dataset; includes 11 open conversions in MIS intention-to-treat group.
Xu et al., 2022	China; 5 centres	Yes	Retrospective multicentre comparative cohort	Pathology-confirmed distal cholangiocarcinoma; no neoadjuvant therapy	dCCA	No	RPD vs OPD	RPD	PSM 180 vs 180	PSM univariable HRs (OS/RFS; reported as OPD vs RPD and inverted in sheet)	1:1 PSM	Large multicentre robotic series from post-learning-curve centres.

Lee et al., 2023	Korea; single tertiary centre	No	Retrospective comparative cohort	Distal bile duct cancer (DBDC), AJCC stage	DBDC/dCCA	Yes	MIPD vs OPD	Mixed-MIPD	Crude actual cohort 81 vs 288 for arm-level RevMan entry	IPTW HRs before mediator adjustment (OS/RFS)	IPTW primary; PSM sensitivity	Stage-restricted DBDC cohort; weighted perioperative tables are available but crude arm-level counts are cleaner for RevMan entry.
Uijterwijk et al., 2023	International multicentre (8 centres, 5 countries)	Yes	Retrospective international comparative cohort	Resected distal cholangiocarcinoma	dCCA	No	MIPD vs OPD	Mixed-MIPD	PSM 97 vs 96	Matched KM summary; direct HR in sheet is from overall cohort	1:1 PSM	Matched OPD controls only; MIPD values unchanged after matching because all 97 MIS cases were retained. Margin reported as R1 <1 mm.
Gao et al., 2025	China; single centre	No	Retrospective comparative cohort	Distal cholangiocarcinoma after surgeon learning curve	dCCA	No	LPD vs OPD	LPD	PSM 184 vs 184	Matched KM summary for OS; no direct arm-level HR in main paper	1:1 PSM	First dCCA paper in this set to report Textbook Outcome; possible institutional overlap with Xu 2022 should be audited before pooling.

Table S3. Outcome definitions, harmonization rules, and study-specific caveats.

outcome_label	data_type	family	default_effect_measure	primary_pooled_set	core_outcome	unit	direction_of_benefit	analysis_note
Abdominal infection	binary	complication	OR	No	No			
Adjuvant treatment	binary	oncology_process	OR	No	No			
Bile leak	binary	pancreatic_specific_complication	OR	No	Yes		lower_favors_mis	
CR-POPF	binary	pancreatic_specific_complication	OR	Yes	Yes		lower_favors_mis	
CR-PPH	binary	pancreatic_specific_complication	OR	No	No			
DGE	binary	pancreatic_specific_complication	OR	No	Yes		lower_favors_mis	
Early mortality	binary	mortality	OR	Yes	Yes		lower_favors_mis	Pool cautiously because definitions may differ across studies (30-day, 90-day, or in-hospital).
Follow-up mortality	binary	survival_descriptive	OR	No	No			
Major morbidity	binary	complication	OR	Yes	Yes		lower_favors_mis	Pool severe postoperative morbidity, recognizing that Uijterwijk 2023 used Clavien-Dindo grade 3b-5 while most other cohorts used grade III or III-IV.
Postoperative complications	binary	complication	OR	No	No			
PPH	binary	pancreatic_specific_complication	OR	No	No			
R0 resection	binary	pathology	OR	Yes	Yes		higher_favors_mis	Pool as reported for the primary analysis; Uijterwijk 2023 also reported an R1 <1 mm margin rule that should be handled in sensitivity analyses.
R1 (<1 mm)	binary	pathology	OR	No	No			
Readmission	binary	resource_use	OR	No	No			
Reoperation	binary	complication	OR	No	No			
SSI	binary	complication	OR	No	No			

Textbook outcome	binary	composite	OR	No	No		higher_favors_mis	Narrative or secondary analysis only unless additional compatible studies become available.
Transfusion	binary	perioperative_resource	OR	No	Yes		lower_favors_mis	
Blood loss (mL)	continuous	intraoperative	MD	Yes	Yes	mL	lower_favors_mis	Use raw mean and SD when reported; use converted statistics only if prespecified and transparently labeled.
Length of stay (days)	continuous	resource_use	MD	Yes	Yes	days	lower_favors_mis	Pool as mean difference in days; clinical pathways may contribute to heterogeneity.
Lymph node yield	continuous	pathology	MD	Yes	Yes	nodes	higher_favors_mis	Pool as mean difference in number of nodes; pathology processing and extent of dissection may contribute to heterogeneity.
Operative time (min)	continuous	intraoperative	MD	Yes	Yes	min	lower_favors_mis	Pool as mean difference in minutes.
DFI	survival	survival	HR	No	No		lower_hazard_favors_mis	Keep separate from DFS/RFS in the primary analysis; consider a recurrence-free family sensitivity analysis only if prespecified.
DFS	survival	survival	HR	No	No		lower_hazard_favors_mis	Keep separate from RFS/DFI in the primary analysis; consider a recurrence-free family sensitivity analysis only if prespecified.
OS	survival	survival	HR	Yes	Yes		lower_hazard_favors_mis	Primary survival endpoint. Pool only direct HRs in the primary analysis; KM-only matched cohorts can be added in sensitivity analyses after digitization.
RFS	survival	survival	HR	No	No		lower_hazard_favors_mis	Keep separate from DFS/DFI in the primary analysis; consider a recurrence-free family sensitivity analysis only if prespecified.

Table S4. Study-level extraction structure for pooled outcomes.

study_id	data_type	analysis_set	outcome	primary_pooled_set	notes
Zhu 2022	Binary	PSM 30 vs 30	R0 resection	Yes	Directly extractable matched counts.
Zhu 2022	Binary	PSM 30 vs 30	Abdominal infection	Yes	Not a universal core endpoint; optional sensitivity.
Zhu 2022	Binary	PSM 30 vs 30	CR-POPF	Yes	
Zhu 2022	Binary	PSM 30 vs 30	DGE	Yes	
Zhu 2022	Binary	PSM 30 vs 30	Follow-up mortality	No	Not for perioperative meta; useful for descriptive survival context.
Kim 2022	Binary	PSM 91 vs 182	Transfusion	Yes	
Kim 2022	Binary	PSM 91 vs 182	R0 resection	Yes	
Kim 2022	Binary	PSM 91 vs 182	Major morbidity	Yes	Primary major morbidity definition differs slightly from Uijterwijk 2023.
Kim 2022	Binary	PSM 91 vs 182	CR-POPF	Yes	
Kim 2022	Binary	PSM 91 vs 182	Early mortality	Yes	Pool with other early mortality definitions using sensitivity analysis.
Xu 2022	Binary	PSM 180 vs 180	Transfusion	Yes	
Xu 2022	Binary	PSM 180 vs 180	R0 resection	Yes	
Xu 2022	Binary	PSM 180 vs 180	Major morbidity	Yes	
Xu 2022	Binary	PSM 180 vs 180	CR-POPF	Yes	Summed from grade B and C rows.
Xu 2022	Binary	PSM 180 vs 180	Bile leak	Yes	
Xu 2022	Binary	PSM 180 vs 180	DGE	Yes	Summed from grade B and C rows.
Xu 2022	Binary	PSM 180 vs 180	Reoperation	Yes	
Xu 2022	Binary	PSM 180 vs 180	Early mortality	Yes	
Lee 2023	Binary	Crude cohort 81 vs 288	Transfusion	Yes	Preferred arm-level dataset uses actual sample sizes rather than weighted pseudo-cohort.
Lee 2023	Binary	Crude cohort 81 vs 288	R0 resection	Yes	Derived from reported R1 counts.
Lee 2023	Binary	Crude cohort 81 vs 288	Major morbidity	Yes	
Lee 2023	Binary	Crude cohort 81 vs 288	CR-POPF	Yes	
Lee 2023	Binary	Crude cohort 81 vs 288	Adjuvant treatment	No	Not a clinical outcome for MIS effect; descriptive only.

Lee 2023	Binary	IPTW weighted cohort 81 vs 286	Transfusion	No	Alternative adjusted arm-level dataset; weighted pseudo-cohort, use cautiously.
Lee 2023	Binary	IPTW weighted cohort 81 vs 286	R0 resection	No	Alternative adjusted arm-level dataset; weighted pseudo-cohort.
Lee 2023	Binary	IPTW weighted cohort 81 vs 286	Major morbidity	No	Alternative adjusted arm-level dataset; weighted pseudo-cohort.
Lee 2023	Binary	IPTW weighted cohort 81 vs 286	CR-POPF	No	Alternative adjusted arm-level dataset; weighted pseudo-cohort.
Uijterwijk 2023	Binary	PSM 97 vs 96	Postoperative complications	Yes	MIPD values unchanged after matching because all 97 MIS cases were retained.
Uijterwijk 2023	Binary	PSM 97 vs 96	Major morbidity	Yes	Definition stricter than most other studies.
Uijterwijk 2023	Binary	PSM 97 vs 96	Early mortality	Yes	
Uijterwijk 2023	Binary	PSM 97 vs 96	CR-POPF	Yes	
Uijterwijk 2023	Binary	PSM 97 vs 96	CR-PPH	No	Optional secondary endpoint.
Uijterwijk 2023	Binary	PSM 97 vs 96	DGE	Yes	
Uijterwijk 2023	Binary	PSM 97 vs 96	Bile leak	Yes	
Uijterwijk 2023	Binary	PSM 97 vs 96	SSI	Yes	
Uijterwijk 2023	Binary	PSM 97 vs 96	R1 (<1 mm)	No	Do not merge with standard R0/R1 studies unless 1-mm rule is prespecified.
Gao 2025	Binary	PSM 184 vs 184	Transfusion	Yes	
Gao 2025	Binary	PSM 184 vs 184	R0 resection	Yes	
Gao 2025	Binary	PSM 184 vs 184	Major morbidity	Yes	
Gao 2025	Binary	PSM 184 vs 184	CR-POPF	Yes	Summed from grade B and C rows.
Gao 2025	Binary	PSM 184 vs 184	Bile leak	Yes	Reported as BL.
Gao 2025	Binary	PSM 184 vs 184	DGE	Yes	Summed from grade B and C rows.
Gao 2025	Binary	PSM 184 vs 184	PPH	No	Optional secondary endpoint.
Gao 2025	Binary	PSM 184 vs 184	Reoperation	Yes	
Gao 2025	Binary	PSM 184 vs 184	Readmission	Yes	
Gao 2025	Binary	PSM 184 vs 184	Early mortality	Yes	
Gao 2025	Binary	PSM 184 vs 184	Textbook outcome	Yes	Novel composite endpoint for secondary analysis or narrative synthesis.

Zhu 2022	Continuous	PSM 30 vs 30	Blood loss (mL)	No	No dispersion reported; not directly poolable unless authors/supplement provide SD/IQR.
Zhu 2022	Continuous	PSM 30 vs 30	Lymph node yield	No	No dispersion reported; not directly poolable unless authors/supplement provide SD/IQR.
Zhu 2022	Continuous	PSM 30 vs 30	Length of stay (days)	No	No dispersion reported; not directly poolable unless authors/supplement provide SD/IQR.
Kim 2022	Continuous	PSM 91 vs 182	Operative time (min)	Yes	
Kim 2022	Continuous	PSM 91 vs 182	Blood loss (mL)	Yes	Converted mean/SD helper columns use Wan/Luo approximation.
Kim 2022	Continuous	PSM 91 vs 182	Lymph node yield	Yes	
Kim 2022	Continuous	PSM 91 vs 182	Length of stay (days)	Yes	
Xu 2022	Continuous	PSM 180 vs 180	Operative time (min)	Yes	Converted mean/SD helper columns use Wan/Luo approximation.
Xu 2022	Continuous	PSM 180 vs 180	Blood loss (mL)	Yes	Converted mean/SD helper columns use Wan/Luo approximation.
Xu 2022	Continuous	PSM 180 vs 180	Lymph node yield	Yes	Converted mean/SD helper columns use Wan/Luo approximation.
Xu 2022	Continuous	PSM 180 vs 180	Length of stay (days)	Yes	Converted mean/SD helper columns use Wan/Luo approximation.
Lee 2023	Continuous	Crude cohort 81 vs 288	Operative time (min)	Yes	Crude actual cohort selected for direct arm-level RevMan entry.
Lee 2023	Continuous	Crude cohort 81 vs 288	Lymph node yield	Yes	Crude actual cohort selected for direct arm-level RevMan entry.
Lee 2023	Continuous	Crude cohort 81 vs 288	Length of stay (days)	Yes	Crude actual cohort selected for direct arm-level RevMan entry.
Lee 2023	Continuous	IPTW weighted cohort 81 vs 286	Operative time (min)	No	Alternative adjusted arm-level dataset; weighted pseudo-cohort.
Lee 2023	Continuous	IPTW weighted cohort 81 vs 286	Lymph node yield	No	Alternative adjusted arm-level dataset; weighted pseudo-cohort.
Lee 2023	Continuous	IPTW weighted cohort 81 vs 286	Length of stay (days)	No	Alternative adjusted arm-level dataset; weighted pseudo-cohort.
Uijterwijk 2023	Continuous	PSM 97 vs 96	Blood loss (mL)	Yes	Matched MIPD values identical to overall MIPD because all 97 MIS patients were retained.
Uijterwijk 2023	Continuous	PSM 97 vs 96	Operative time (min)	Yes	Matched MIPD values identical to overall MIPD because all 97 MIS patients were retained.
Uijterwijk 2023	Continuous	PSM 97 vs 96	Length of stay (days)	Yes	Matched MIPD values identical to overall MIPD because all 97 MIS patients were retained.

Uijterwijk 2023	Continuous	PSM 97 vs 96	Lymph node yield	Yes	Matched MIPD values identical to overall MIPD because all 97 MIS patients were retained.
Gao 2025	Continuous	PSM 184 vs 184	Operative time (min)	Yes	Converted mean/SD helper columns use Wan/Luo approximation.
Gao 2025	Continuous	PSM 184 vs 184	Blood loss (mL)	Yes	Converted mean/SD helper columns use Wan/Luo approximation.
Gao 2025	Continuous	PSM 184 vs 184	Lymph node yield	Yes	Converted mean/SD helper columns use Wan/Luo approximation.
Gao 2025	Continuous	PSM 184 vs 184	Length of stay (days)	Yes	Converted mean/SD helper columns use Wan/Luo approximation.
Zhu 2022	Survival	Overall cohort univariable Cox	OS	No	Direct HR available, but primary matched analysis in paper is KM only.
Zhu 2022	Survival	Overall cohort multivariable Cox	OS	No	Very wide CI; likely overfit. Not recommended for primary pooled estimate.
Zhu 2022	Survival	PSM KM summary	OS	No	Primary survival comparison in matched cohort is KM only.
Kim 2022	Survival	PSM multivariable Cox	DFS	Yes	
Kim 2022	Survival	PSM multivariable Cox	OS	Yes	
Xu 2022	Survival	PSM univariable Cox	OS	Yes	Main-text survival HR reported in opposite direction; sheet auto-inverts to MIS vs Open.
Xu 2022	Survival	PSM univariable Cox	RFS	Yes	Main-text survival HR reported in opposite direction; sheet auto-inverts to MIS vs Open.
Lee 2023	Survival	IPTW before mediator adjustment	OS	Yes	Preferred total-effect estimate; does not adjust for postoperative mediators.
Lee 2023	Survival	IPTW before mediator adjustment	RFS	Yes	Preferred total-effect estimate; does not adjust for postoperative mediators.
Lee 2023	Survival	IPTW after mediator adjustment	OS	No	Use only for sensitivity analysis; postoperative factors may be mediators.
Lee 2023	Survival	IPTW after mediator adjustment	RFS	No	Use only for sensitivity analysis; postoperative factors may be mediators.
Lee 2023	Survival	PSM sensitivity before mediator adjustment	OS	No	Sensitivity analysis in matched cohort.
Lee 2023	Survival	PSM sensitivity before mediator adjustment	RFS	No	Sensitivity analysis in matched cohort.
Lee 2023	Survival	PSM sensitivity after mediator adjustment	OS	No	Sensitivity analysis; mediator-adjusted.
Lee 2023	Survival	PSM sensitivity after mediator adjustment	RFS	No	Sensitivity analysis; mediator-adjusted.

Uijterwijk 2023	Survival	Overall cohort univariable Cox	OS	No	Direct HR available only from overall cohort; matched analysis reported as KM summary.
Uijterwijk 2023	Survival	Overall cohort univariable Cox	DFI	No	Direct HR available only from overall cohort; matched analysis reported as KM summary.
Uijterwijk 2023	Survival	PSM KM summary	OS	No	KM extraction required if matched HR is desired.
Uijterwijk 2023	Survival	PSM KM summary	DFI	No	KM extraction required if matched HR is desired.
Gao 2025	Survival	PSM KM summary	OS	No	No direct arm-level HR in main paper; KM extraction required.
Rows marked "No" in the primary pooled set remain useful for sensitivity, narrative, or future network analyses.					

Table S5. Descriptive heterogeneity summary: study-level characteristics and directional effects.

Study	Country / Setting	Platform	Adjustment	N (MIS vs Open)	Blood loss direction	R0 direction	OS direction	Heterogeneity notes
Zhu 2022	China, single center	LPD	1:1 PSM	30 vs 30	Favors MIS*	Neutral (28/30 vs 28/30)	Favors MIS (KM only)	No dispersion; not poolable for continuous
Kim 2022	Korea, 2 tertiary centers	Mixed MIPD	1:2 PSM	91 vs 182	Favors MIS	Favors MIS (87/91 vs 169/182)	Favors MIS (HR 0.89)	Largest matched cohort; includes 11 conversions
Xu 2022	China, 5 centers	RPD	1:1 PSM	180 vs 180	Favors MIS	Favors MIS (161/180 vs 154/180)	Neutral (HR 1.02)	Post-learning-curve robotic series
Lee 2023	Korea, single center	Mixed MIPD	IPTW primary	81 vs 288	Not pooled	Neutral (crude); Favors MIS (IPTW)	Favors MIS (HR 0.60)	Stage I–IIb only; crude vs IPTW sensitivity
Uijterwijk 2023	International, 8 centers	Mixed MIPD	1:1 PSM	97 vs 96	Favors MIS	1-mm rule (excluded from primary R0)	Neutral (reconstructed HR 0.87)	Only non–East Asian cohort; R1<1mm margin
Gao 2025	China, single center	LPD	1:1 PSM	184 vs 184	Favors MIS	Neutral (167/184 vs 167/184)	Favors MIS (reconstructed HR 0.81)	Post-learning-curve; reports Textbook Outcome

* Blood loss reported without dispersion measures; direction based on mean values only. This table is intended as a descriptive overview of study-level heterogeneity sources and should not be interpreted as a formal subgroup analysis. LPD, laparoscopic pancreaticoduodenectomy; RPD, robotic pancreaticoduodenectomy; MIPD, minimally invasive pancreaticoduodenectomy; PSM, propensity-score matching; IPTW, inverse probability of treatment weighting.

Table S6. Binary outcome input table.

study_id	mis_label	analysis_set	outcome_label	mis_events	mis_total	open_events	open_total	effect_measure	primary_pooled_set	notes
Zhu 2022	LPD	PSM 30 vs 30	R0 resection	28	30	28	30	OR	TRUE	Directly extractable matched counts.
Zhu 2022	LPD	PSM 30 vs 30	Abdominal infection	5	30	4	30	OR	TRUE	Not a universal core endpoint; optional sensitivity.
Zhu 2022	LPD	PSM 30 vs 30	CR-POPF	8	30	8	30	OR	TRUE	
Zhu 2022	LPD	PSM 30 vs 30	DGE	2	30	4	30	OR	TRUE	

Zhu 2022	LPD	PSM 30 vs 30	Follow-up mortality	5	30	22	30	OR	FALSE	Not for perioperative meta; useful for descriptive survival context.
Kim 2022	MIPD (83 laparoscopic + 8 robotic)	PSM 91 vs 182	Transfusion	3	91	17	182	OR	TRUE	
Kim 2022	MIPD (83 laparoscopic + 8 robotic)	PSM 91 vs 182	R0 resection	87	91	169	182	OR	TRUE	
Kim 2022	MIPD (83 laparoscopic + 8 robotic)	PSM 91 vs 182	Major morbidity	24	91	38	182	OR	TRUE	Primary major morbidity definition differs slightly from Uijterwijk 2023.
Kim 2022	MIPD (83 laparoscopic + 8 robotic)	PSM 91 vs 182	CR-POPF	17	91	35	182	OR	TRUE	
Kim 2022	MIPD (83 laparoscopic + 8 robotic)	PSM 91 vs 182	Early mortality	0	91	3	182	OR	TRUE	Pool with other early mortality definitions using sensitivity analysis.
Xu 2022	RPD	PSM 180 vs 180	Transfusion	7	180	8	180	OR	TRUE	
Xu 2022	RPD	PSM 180 vs 180	R0 resection	161	180	154	180	OR	TRUE	
Xu 2022	RPD	PSM 180 vs 180	Major morbidity	28	180	30	180	OR	TRUE	
Xu 2022	RPD	PSM 180 vs 180	CR-POPF	18	180	16	180	OR	TRUE	Summed from grade B and C rows.
Xu 2022	RPD	PSM 180 vs 180	Bile leak	10	180	11	180	OR	TRUE	
Xu 2022	RPD	PSM 180 vs 180	DGE	22	180	21	180	OR	TRUE	Summed from grade B and C rows.
Xu 2022	RPD	PSM 180 vs 180	Reoperation	7	180	7	180	OR	TRUE	
Xu 2022	RPD	PSM 180 vs 180	Early mortality	5	180	6	180	OR	TRUE	

Lee 2023	MIPD (60 laparoscopic + 21 robotic)	Crude cohort 81 vs 288	Transfusion	11	81	33	288	OR	TRUE	Preferred arm-level dataset uses actual sample sizes rather than weighted pseudo-cohort.
Lee 2023	MIPD (60 laparoscopic + 21 robotic)	Crude cohort 81 vs 288	R0 resection	67	81	233	288	OR	TRUE	Derived from reported R1 counts.
Lee 2023	MIPD (60 laparoscopic + 21 robotic)	Crude cohort 81 vs 288	Major morbidity	6	81	39	288	OR	TRUE	
Lee 2023	MIPD (60 laparoscopic + 21 robotic)	Crude cohort 81 vs 288	CR-POPF	9	81	24	288	OR	TRUE	
Lee 2023	MIPD (60 laparoscopic + 21 robotic)	Crude cohort 81 vs 288	Adjuvant treatment	46	81	194	288	OR	FALSE	Not a clinical outcome for MIS effect; descriptive only.
Lee 2023	MIPD (60 laparoscopic + 21 robotic)	IPTW weighted cohort 81 vs 286	Transfusion	14	81	33	286	OR	FALSE	Alternative adjusted arm-level dataset; weighted pseudo-cohort, use cautiously.
Lee 2023	MIPD (60 laparoscopic + 21 robotic)	IPTW weighted cohort 81 vs 286	R0 resection	69	81	231	286	OR	FALSE	Alternative adjusted arm-level dataset; weighted pseudo-cohort.
Lee 2023	MIPD (60 laparoscopic + 21 robotic)	IPTW weighted cohort 81 vs 286	Major morbidity	7	81	38	286	OR	FALSE	Alternative adjusted arm-level dataset; weighted pseudo-cohort.
Lee 2023	MIPD (60 laparoscopic + 21 robotic)	IPTW weighted cohort 81 vs 286	CR-POPF	9	81	24	286	OR	FALSE	Alternative adjusted arm-level dataset; weighted pseudo-cohort.

Uijterwijk 2023	MIPD (60 LPD + 37 RPD + 7 hybrid)	PSM 97 vs 96	Postoperative complications	64	97	79	96	OR	TRUE	MIPD values unchanged after matching because all 97 MIS cases were retained.
Uijterwijk 2023	MIPD (60 LPD + 37 RPD + 7 hybrid)	PSM 97 vs 96	Major morbidity	22	97	19	96	OR	TRUE	Definition stricter than most other studies.
Uijterwijk 2023	MIPD (60 LPD + 37 RPD + 7 hybrid)	PSM 97 vs 96	Early mortality	3	97	2	96	OR	TRUE	
Uijterwijk 2023	MIPD (60 LPD + 37 RPD + 7 hybrid)	PSM 97 vs 96	CR-POPF	26	97	24	96	OR	TRUE	
Uijterwijk 2023	MIPD (60 LPD + 37 RPD + 7 hybrid)	PSM 97 vs 96	CR-PPH	16	97	14	96	OR	FALSE	Optional secondary endpoint.
Uijterwijk 2023	MIPD (60 LPD + 37 RPD + 7 hybrid)	PSM 97 vs 96	DGE	25	97	30	96	OR	TRUE	
Uijterwijk 2023	MIPD (60 LPD + 37 RPD + 7 hybrid)	PSM 97 vs 96	Bile leak	10	97	12	96	OR	TRUE	
Uijterwijk 2023	MIPD (60 LPD + 37 RPD + 7 hybrid)	PSM 97 vs 96	SSI	7	97	17	96	OR	TRUE	
Uijterwijk 2023	MIPD (60 LPD + 37 RPD + 7 hybrid)	PSM 97 vs 96	R1 (<1 mm)	23	97	29	96	OR	FALSE	Do not merge with standard R0/R1 studies unless 1-mm rule is prespecified.
Gao 2025	LPD	PSM 184 vs 184	Transfusion	14	184	16	184	OR	TRUE	
Gao 2025	LPD	PSM 184 vs 184	R0 resection	167	184	167	184	OR	TRUE	
Gao 2025	LPD	PSM 184 vs 184	Major morbidity	25	184	30	184	OR	TRUE	
Gao 2025	LPD	PSM 184 vs 184	CR-POPF	19	184	23	184	OR	TRUE	Summed from grade B and C rows.
Gao 2025	LPD	PSM 184 vs 184	Bile leak	17	184	14	184	OR	TRUE	Reported as BL.

Gao 2025	LPD	PSM 184 vs 184	DGE	22	184	23	184	OR	TRUE	Summed from grade B and C rows.
Gao 2025	LPD	PSM 184 vs 184	PPH	14	184	13	184	OR	FALSE	Optional secondary endpoint.
Gao 2025	LPD	PSM 184 vs 184	Reoperation	6	184	4	184	OR	TRUE	
Gao 2025	LPD	PSM 184 vs 184	Readmission	6	184	10	184	OR	TRUE	
Gao 2025	LPD	PSM 184 vs 184	Early mortality	3	184	4	184	OR	TRUE	
Gao 2025	LPD	PSM 184 vs 184	Textbook outcome	115	184	111	184	OR	TRUE	Novel composite endpoint for secondary analysis or narrative synthesis.

Table S7. Continuous outcome input table.

study_id	mis_label	analysis_set	outcome_label	mis_total	mis_mean	mis_sd	open_total	open_mean	open_sd	effect_measure	primary_pooled_set	notes
Zhu 2022	LPD	PSM 30 vs 30	Blood loss (mL)	30	302.33		30	505		MD	FALSE	No dispersion reported; not directly poolable unless authors/supplement provide SD/IQR.
Zhu 2022	LPD	PSM 30 vs 30	Lymph node yield	30	14.3		30	9.93		MD	FALSE	No dispersion reported; not directly poolable unless authors/supplement provide SD/IQR.
Zhu 2022	LPD	PSM 30 vs 30	Length of stay (days)	30	25.1		30	24.03		MD	FALSE	No dispersion reported; not directly poolable unless authors/supplement provide SD/IQR.
Kim 2022	MIPD (83 laparoscopic + 8 robotic)	PSM 91 vs 182	Operative time (min)	91	457	70	182	398	85	MD	TRUE	
Kim 2022	MIPD (83 laparoscopic + 8 robotic)	PSM 91 vs 182	Blood loss (mL)	91	266.67	185.19	182	400	296.3	MD	TRUE	Converted mean/SD helper columns use Wan/Luo approximation.
Kim 2022	MIPD (83 laparoscopic + 8 robotic)	PSM 91 vs 182	Lymph node yield	91	14.9	7.8	182	20.7	11.5	MD	TRUE	
Kim 2022	MIPD (83 laparoscopic + 8 robotic)	PSM 91 vs 182	Length of stay (days)	91	19.8	11.3	182	26.6	14.3	MD	TRUE	
Xu 2022	RPD	PSM 180 vs 180	Operative time (min)	180	269.17	46.3	180	263	45.93	MD	TRUE	Converted mean/SD helper columns use Wan/Luo approximation.

Xu 2022	RPD	PSM 180 vs 180	Blood loss (mL)	180	150	74.07	180	250	222.22	MD	TRUE	Converted mean/SD helper columns use Wan/Luo approximation.
Xu 2022	RPD	PSM 180 vs 180	Lymph node yield	180	13.67	4.44	180	12.67	3.7	MD	TRUE	Converted mean/SD helper columns use Wan/Luo approximation.
Xu 2022	RPD	PSM 180 vs 180	Length of stay (days)	180	13	5.19	180	15	4.44	MD	TRUE	Converted mean/SD helper columns use Wan/Luo approximation.
Lee 2023	MIPD (60 laparoscopic + 21 robotic)	Crude cohort 81 vs 288	Operative time (min)	81	366.2	92.8	288	279.1	66.9	MD	TRUE	Crude actual cohort selected for direct arm-level RevMan entry.
Lee 2023	MIPD (60 laparoscopic + 21 robotic)	Crude cohort 81 vs 288	Lymph node yield	81	17.2	6.7	288	18.7	7.7	MD	TRUE	Crude actual cohort selected for direct arm-level RevMan entry.
Lee 2023	MIPD (60 laparoscopic + 21 robotic)	Crude cohort 81 vs 288	Length of stay (days)	81	12	4	288	12	3	MD	TRUE	Crude actual cohort selected for direct arm-level RevMan entry.
Lee 2023	MIPD (60 laparoscopic + 21 robotic)	IPTW weighted cohort 81 vs 286	Operative time (min)	81	364.2	88.9	286	278	66.3	MD	FALSE	Alternative adjusted arm-level dataset; weighted pseudo-cohort.
Lee 2023	MIPD (60 laparoscopic + 21 robotic)	IPTW weighted cohort 81 vs 286	Lymph node yield	81	17.2	6.3	286	18.7	7.7	MD	FALSE	Alternative adjusted arm-level dataset; weighted pseudo-cohort.
Lee 2023	MIPD (60 laparoscopic + 21 robotic)	IPTW weighted cohort 81 vs 286	Length of stay (days)	81	12	4	286	12	3	MD	FALSE	Alternative adjusted arm-level dataset; weighted pseudo-cohort.

Uijterwijk 2023	MIPD (60 LPD + 37 RPD + 7 hybrid)	PSM 97 vs 96	Blood loss (mL)	97	333.33	222.22	96	498.33	388.89	MD	TRUE	Matched MIPD values identical to overall MIPD because all 97 MIS patients were retained.
Uijterwijk 2023	MIPD (60 LPD + 37 RPD + 7 hybrid)	PSM 97 vs 96	Operative time (min)	97	459.67	130.37	96	340	115.56	MD	TRUE	Matched MIPD values identical to overall MIPD because all 97 MIS patients were retained.
Uijterwijk 2023	MIPD (60 LPD + 37 RPD + 7 hybrid)	PSM 97 vs 96	Length of stay (days)	97	18.33	13.33	96	20.33	15.56	MD	TRUE	Matched MIPD values identical to overall MIPD because all 97 MIS patients were retained.
Uijterwijk 2023	MIPD (60 LPD + 37 RPD + 7 hybrid)	PSM 97 vs 96	Lymph node yield	97	16	8.89	96	14.67	5.93	MD	TRUE	Matched MIPD values identical to overall MIPD because all 97 MIS patients were retained.
Gao 2025	LPD	PSM 184 vs 184	Operative time (min)	184	303.33	103.7	184	304	105.93	MD	TRUE	Converted mean/SD helper columns use Wan/Luo approximation.
Gao 2025	LPD	PSM 184 vs 184	Blood loss (mL)	184	163.33	155.56	184	250	222.22	MD	TRUE	Converted mean/SD helper columns use Wan/Luo approximation.
Gao 2025	LPD	PSM 184 vs 184	Lymph node yield	184	13.67	6.67	184	13.67	5.93	MD	TRUE	Converted mean/SD helper columns use Wan/Luo approximation.

Gao 2025	LPD	PSM 184 vs 184	Length of stay (days)	184	13.33	5.19	184	15.33	4.44	MD	TRUE	Converted mean/SD helper columns use Wan/Luo approximation.

Table S8. Direct survival input table.

study_id	mis_subgroup	analysis_set	outcome_label	hr	lower_95_ci	upper_95_ci	log_hr	se_log_hr	primary_pooled_set	participant_total	notes
Zhu 2022	LPD	Overall cohort univariable Cox	OS	0.46	0.25	0.85	-0.77	0.31	FALSE	60	Direct HR available, but primary matched analysis in paper is KM only.
Zhu 2022	LPD	Overall cohort multivariable Cox	OS	0.26	0.04	1.87	-1.35	1.01	FALSE	60	Very wide CI; likely overfit. Not recommended for primary pooled estimate.
Zhu 2022	LPD	PSM KM summary	OS						FALSE	60	Primary survival comparison in matched cohort is KM only.
Kim 2022	Mixed-MIPD	PSM multivariable Cox	DFS	0.93	0.61	1.43	-0.07	0.22	TRUE	273	
Kim 2022	Mixed-MIPD	PSM multivariable Cox	OS	0.89	0.57	1.37	-0.12	0.22	TRUE	273	
Xu 2022	RPD	PSM univariable Cox	OS	1.02	0.78	1.35	0.02	0.14	TRUE	360	Main-text survival HR reported in opposite direction; sheet auto-inverts to MIS vs Open.
Xu 2022	RPD	PSM univariable Cox	RFS	0.92	0.71	1.20	-0.08	0.13	TRUE	360	Main-text survival HR reported in opposite direction; sheet auto-inverts to MIS vs Open.
Lee 2023	Mixed-MIPD	IPTW before mediator adjustment	OS	0.60	0.30	1.20	-0.51	0.35	TRUE	369	Preferred total-effect estimate; does not adjust for postoperative mediators.

Lee 2023	Mixed-MIPD	IPTW before mediator adjustment	RFS	1.02	0.68	1.53	0.02	0.21	TRUE	369	Preferred total-effect estimate; does not adjust for postoperative mediators.
Lee 2023	Mixed-MIPD	IPTW after mediator adjustment	OS	0.61	0.29	1.26	-0.49	0.37	FALSE	369	Use only for sensitivity analysis; postoperative factors may be mediators.
Lee 2023	Mixed-MIPD	IPTW after mediator adjustment	RFS	1.01	0.67	1.53	0.01	0.21	FALSE	369	Use only for sensitivity analysis; postoperative factors may be mediators.
Lee 2023	Mixed-MIPD	PSM sensitivity before mediator adjustment	OS	0.64	0.31	1.32	-0.45	0.37	FALSE	369	Sensitivity analysis in matched cohort.
Lee 2023	Mixed-MIPD	PSM sensitivity before mediator adjustment	RFS	1.16	0.72	1.92	0.15	0.25	FALSE	369	Sensitivity analysis in matched cohort.
Lee 2023	Mixed-MIPD	PSM sensitivity after mediator adjustment	OS	0.68	0.32	1.44	-0.39	0.38	FALSE	369	Sensitivity analysis; mediator-adjusted.
Lee 2023	Mixed-MIPD	PSM sensitivity after mediator adjustment	RFS	1.12	0.67	1.88	0.11	0.26	FALSE	369	Sensitivity analysis; mediator-adjusted.
Uijterwijk 2023	Mixed-MIPD	Overall cohort univariable Cox	OS	0.92	0.66	1.28	-0.09	0.17	FALSE	193	Direct HR available only from overall cohort; matched analysis reported as KM summary.

Uijterwijk 2023	Mixed-MIPD	Overall cohort univariable Cox	DFI	0.87	0.63	1.21	-0.14	0.17	FALSE	193	Direct HR available only from overall cohort; matched analysis reported as KM summary.
Uijterwijk 2023	Mixed-MIPD	PSM KM summary	OS						FALSE	193	KM extraction required if matched HR is desired.
Uijterwijk 2023	Mixed-MIPD	PSM KM summary	DFI						FALSE	193	KM extraction required if matched HR is desired.
Gao 2025	LPD	PSM KM summary	OS						FALSE	368	No direct arm-level HR in main paper; KM extraction required.

Table S9. GRADE-based certainty assessment for key outcomes.

Outcome	No. of participants (studies)	Risk of bias	Inconsistency	Indirectness	Imprecision	Reporting bias	Certainty of evidence
Blood loss	1194 (4)	Not serious	Not serious	Not serious	Not serious	Not serious	Low (⊕⊕⊖⊖)
CR-POPF	1623 (6)	Not serious	Not serious	Not serious	Serious a	High risk b	Very Low (⊕⊖⊖⊖)
Major morbidity	1563 (5)	Not serious	Not serious	Not serious	Serious a	High risk b	Very Low (⊕⊖⊖⊖)
R0 resection	1430 (5)	Not serious	Not serious	Not serious	Serious a	High risk b	Very Low (⊕⊖⊖⊖)
Overall survival	1002 (3)	Not serious	Not serious	Not serious	Serious a	High risk b	Very Low (⊕⊖⊖⊖)

a Downgraded 1 level for Serious Imprecision (Domain 4): This is an independent domain downgrade resulting directly from wide confidence intervals that cross the threshold of clinical equivalence for these specific endpoints.
b Downgraded 1 level for High Risk of Reporting Bias (Domain 5): This is an independent domain downgrade reflecting the potential for publication and language bias in this small, English-language restricted, retrospective evidence base, evaluated qualitatively because formal funnel plot testing was underpowered.

Table S10. Pre-defined interpretability framework.

issue	studies_affected	why_it_matters	handling_under_a_priori_interpretability_framework
Margin-definition heterogeneity	Uijterwijk 2023 versus standard-margin cohorts	Uijterwijk 2023 defined R1 as tumor within 1 mm, whereas the primary pooled R0 estimate used broadly comparable definitions.	Primary R0 pooling retained broadly comparable definitions; the 1-mm margin-rule cohort was displayed separately in Figure S13.
Stage-restricted cohort	Lee 2023	Lee 2023 included AJCC stage I to IIb disease only and may not represent the full distal cholangiocarcinoma spectrum.	Retained with explicit interpretability caveats; leave-one-out results were retained in Table S13.
Mixed minimally invasive node	Kim 2022; Uijterwijk 2023	These cohorts grouped laparoscopic and robotic cases within a single MIS node, limiting platform-specific inference.	Retained for MIS versus open pairwise analyses; no formal platform subgroup meta-analysis was performed.
Matched-cohort Kaplan-Meier-only survival	Zhu 2022; Uijterwijk 2023; Gao 2025	Preferred matched or weighted cohorts provided Kaplan-Meier panels without directly reported hazard ratios.	Primary OS pooled model used directly reported HRs only; eligible reconstructed HRs were restricted to the pre-planned sensitivity analysis.
Possible cohort overlap	Xu 2022 and Gao 2025	The two Chinese distal cholangiocarcinoma pancreaticoduodenectomy cohorts had partially overlapping recruitment windows.	Overlap audit was completed before interpretation, and overlap-sensitive reruns were reported without excluding either cohort from the primary models.
Continuous outcomes without dispersion	Zhu 2022	Several continuous outcomes were reported without SD or IQR and were therefore not directly poolable.	Narrative reporting was used when dispersion was unavailable; no SD imputation was introduced.
Early mortality definition heterogeneity	Kim 2022; Xu 2022; Uijterwijk 2023; Gao 2025	Definitions may mix 30-day, 90-day, or in-hospital mortality across studies.	Pooled as a secondary endpoint with an explicit caveat regarding definition heterogeneity.
Conversions and hybrid procedures	Kim 2022; Uijterwijk 2023	Kim 2022 retained conversions in the MIS group and Uijterwijk 2023 included hybrid procedures.	Retained with explicit clinical heterogeneity notes in the interpretability framework.

Table S11. Cohort-screening overlap audit and adjudication notes.

study_a	study_b	country	recruitment_window_a	recruitment_window_b	shared_centers_or_authors_screen	initial_risk	manual_adjudication	final_handling	audit_statement
Xu 2022	Gao 2025	China	2014-2019	2017-2022	Partial recruitment-window overlap and limited author overlap; center sets adjudicated as different.	Possible overlap	Reviewed using country, recruitment window, center descriptors, author overlap, and comparison family.	Retained in primary analyses; overlap-sensitive reruns performed.	No confirmed center-level duplication was identified.
Xu 2022	Zhu 2022	China	2014-2019	2010-2021	No clear shared-center signal identified.	Low overlap concern	Reviewed using country, recruitment window, center descriptors, author overlap, and comparison family.	Retained in primary analyses.	No confirmed center-level duplication was identified.
Gao 2025	Zhu 2022	China	2017-2022	2010-2021	No clear shared-center signal identified.	Low overlap concern	Reviewed using country, recruitment window, center descriptors, author overlap, and comparison family.	Retained in primary analyses.	No confirmed center-level duplication was identified.

Table S12. Kaplan-Meier digitization and reconstruction log.

study_id	preferred_analysis_set	outcome	arm_structure	participant_total	usable_numbers_at_risk	eligible_under_a_priori_interpretability_framework	included_in_pre_planned_sensitivity_analysis	panel_file	coordinate_files	digitization_workflow	reconstructed_hr	lower_95_ci	upper_95_ci	note
Gao 2025	PSM 184 vs 184	OS	MIS/Open	184 vs 184	Yes	Yes	Yes	gao2025_psm_os_panel.png	gao_2025_psm_184_vs_184_os_mis_auto.csv | gao_2025_psm_184_vs_184_os_open_auto.csv	Two investigators independently digitized each eligible panel and checked arm assignment, axis calibration, and pooling eligibility.	0.81	0.62	1.05	Matched-cohort reconstructed HR retained in Figure S11.
Uijterwijk 2023	Matched 97 vs 96	OS	MIS/Open	97 vs 96	Yes	Yes	Yes	uijterwijk2023_matched_os_panel.png	uijterwijk_2023_matched_97_vs_96_os_mis_auto.csv | uijterwijk_2023_matched_97_vs_96_os_open_auto.csv	Two investigators independently digitized each eligible panel and checked arm assignment, axis calibration, and pooling eligibility.	0.87	0.57	1.31	Matched-cohort reconstructed HR retained in Figure S11.
Zhu 2022	PSM 30 vs 30	OS	MIS/Open	30 vs 30	No	No	No	zhu2022_psm_os_panel.png	zhu_2022_psm_30_vs_30_os_mis_auto.csv | zhu_2022_psm_30_vs_30_os_open_auto.csv	Two investigators independently reviewed the panel; reconstruction was not pooled because no usable numbers-at-risk				Matched-cohort panel reviewed only; not pooled in the pre-planned

										table was available.				sensit ivity analy sis.

Table S13. Newcastle-Ottawa Scale scoring.

study_id	exposed_cohort_representative	non_exposed_cohort_selected	exposure_ascertained	outcome_absent_at_baseline	comparability_stage_severity	comparability_other_factors	outcome_assessment	follow_up_long_enough	follow_up_adequate	total_stars_max_9	overall_quality	reviewer_note
Gao 2025	*	*	*	*	*	*	*	*		8	High	Single-centre post-learning-curve cohort with 1:1 PSM and objective outcome assessment; follow-up completeness not fully detailed.
Kim 2022	*	*	*	*	*	*	*	*	*	9	High	Two-centre retrospective cohort with 1:2 PSM, intention-to-treat handling of conversions, and mature perioperative /oncologic follow-up.
Lee 2023	*	*	*	*	*	*	*	*		8	High	Single-centre stage;IIb DBDC cohort with IPTW primary adjustment and PSM sensitivity analysis; follow-up completeness not fully explicit in main text.
Uijterwijk 2023	*	*	*	*	*	*	*	*		8	High	International multicentre cohort with protocol registration and PSM; follow-up

												adequacy not completely described in the main accepted manuscript.
Xu 2022	*	*	*	*	*	*	*	*	*	9	High	Large five-centre propensity score-matched cohort with consecutive patients and explicit eligibility criteria.
Zhu 2022	*	*	*	*	*	*	*	*		8	High	Single-centre retrospective cohort with 1:1 PSM and objective outcomes; incomplete baseline/follow-up data led to exclusions, so follow-up adequacy was not fully awardable.

Table S14. Leave-one-out and overlap-sensitive rerun summary.

endpoint	rerun_category	omitted_study	studies_k	participants	effect_measure	effect_with_ci	i2
Blood loss	reference	None	4	1194	MD	-104.9 (-145.3 to -64.6)	16.3
Blood loss	leave-one-out	Kim 2022	3	921	MD	-99.7 (-160.1 to -39.2)	19.1
Blood loss	overlap-sensitive	Xu 2022	3	834	MD	-116.9 (-211.1 to -22.7)	41.9
Blood loss	leave-one-out	Uijterwijk 2023	3	1001	MD	-100.8 (-148.9 to -52.7)	0.0
Blood loss	overlap-sensitive	Gao 2025	3	826	MD	-117.9 (-189.0 to -46.8)	14.0
R0 resection	reference	None	5	1430	OR	1.22 (0.96 to 1.56)	0
R0 resection	leave-one-out	Zhu 2022	4	1370	OR	1.23 (0.89 to 1.69)	0
R0 resection	leave-one-out	Kim 2022	4	1157	OR	1.18 (0.90 to 1.55)	0
R0 resection	overlap-sensitive	Xu 2022	4	1070	OR	1.13 (0.84 to 1.54)	0
R0 resection	leave-one-out	Lee 2023	4	1061	OR	1.26 (0.87 to 1.82)	0
R0 resection	overlap-sensitive	Gao 2025	4	1062	OR	1.31 (0.99 to 1.73)	0
R0 resection	structure-sensitive	Lee 2023 (IPTW pseudo-cohort replacement)	5	1428	OR	1.25 (1.04 to 1.52)	0
Major morbidity	reference	None	5	1563	OR	0.96 (0.64 to 1.43)	0
Major morbidity	structure-sensitive	Lee 2023 (IPTW pseudo-cohort replacement)	5	1561	OR	0.94 (0.63 to 1.41)	0
Overall survival sensitivity model	reference	None	5	1563	HR	0.88 (0.73 to 1.05)	0
Overall survival sensitivity model	leave-one-out	Kim 2022	4	1290	HR	0.88 (0.68 to 1.14)	0
Overall survival sensitivity model	overlap-sensitive	Xu 2022	4	1203	HR	0.82 (0.68 to 0.97)	0
Overall survival sensitivity model	leave-one-out	Lee 2023	4	1194	HR	0.90 (0.75 to 1.08)	0
Overall survival sensitivity model	overlap-sensitive	Gao 2025	4	1195	HR	0.92 (0.70 to 1.20)	0
Overall survival sensitivity model	leave-one-out	Uijterwijk 2023	4	1370	HR	0.88 (0.68 to 1.14)	0

5. Embedded Supplementary Figures

Figure S1. Survival data evidence hierarchy and reconstruction workflow.

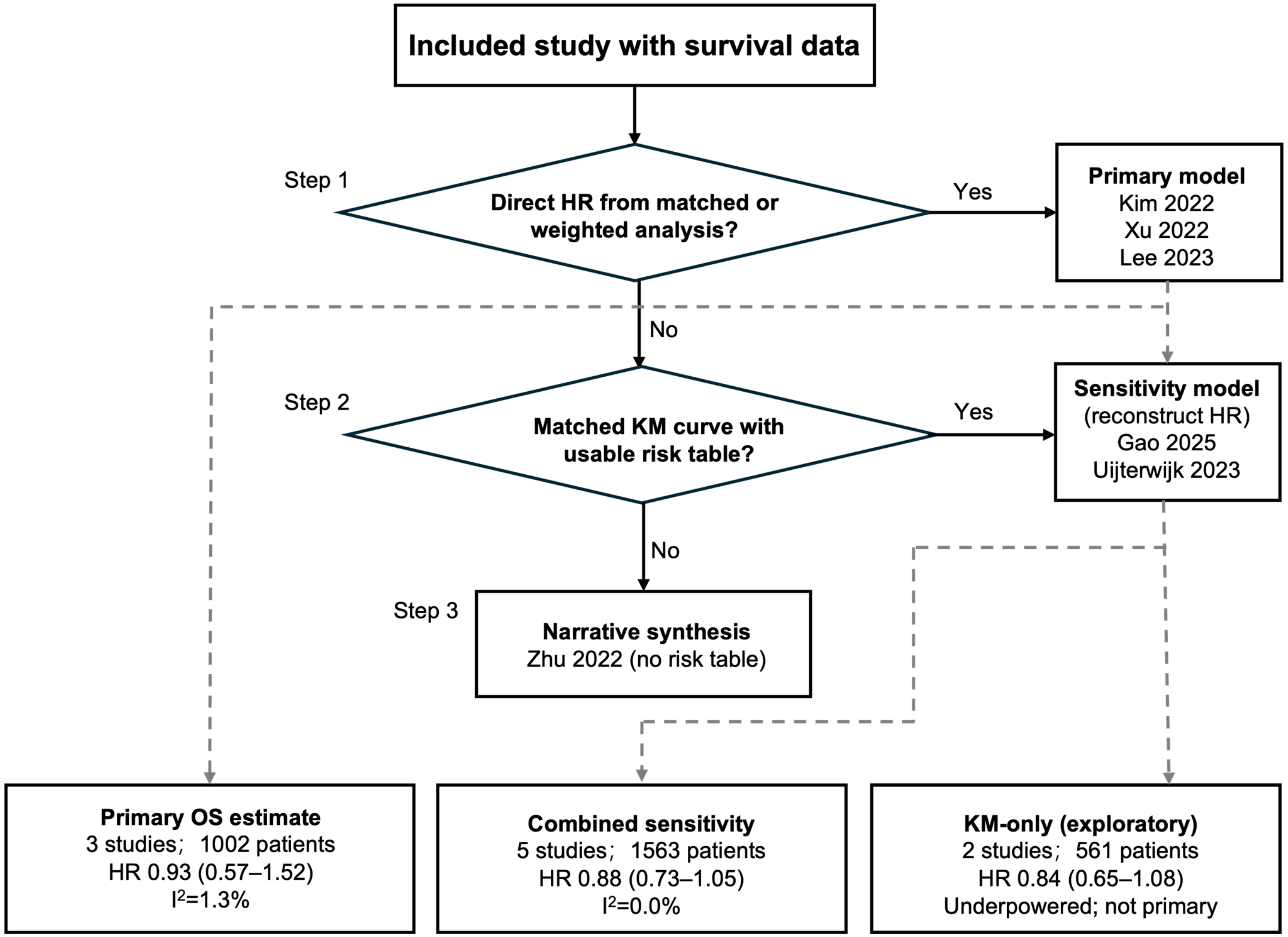

Figure S2. Newcastle-Ottawa Scale heatmap.

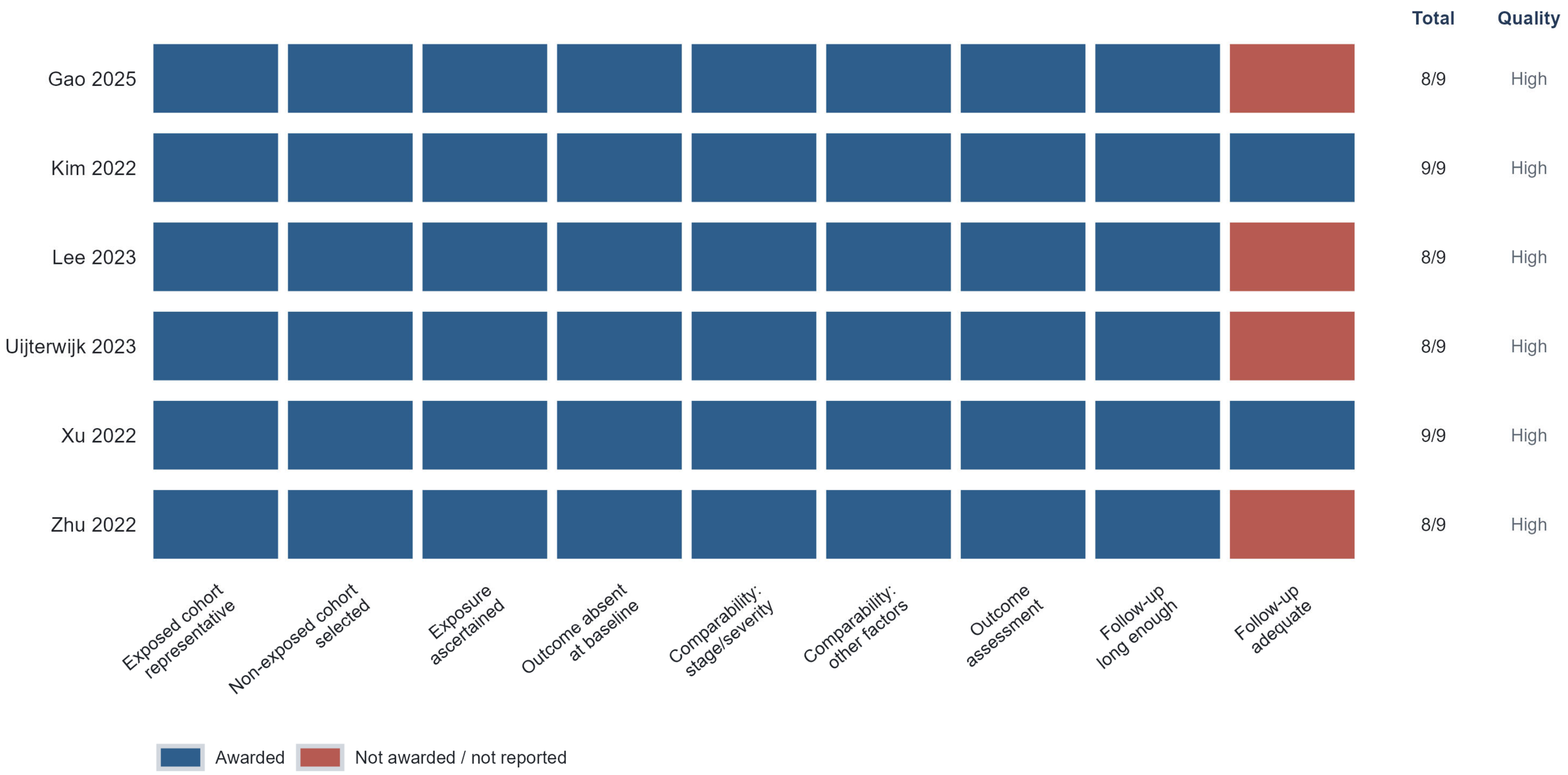

Figure S3. Overlap-sensitive reruns for overall survival.

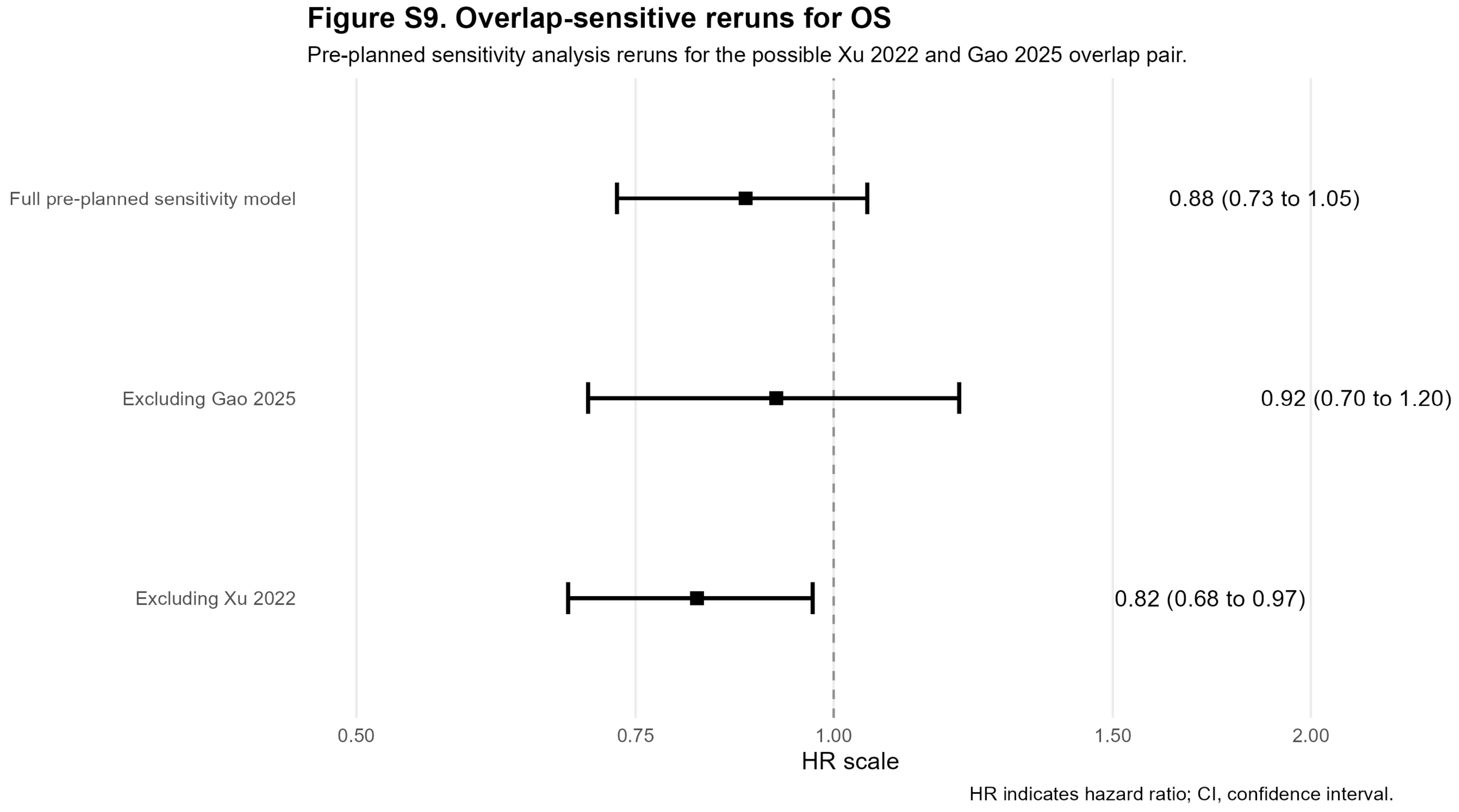

Figure S4. Major morbidity forest plot.

Random-effects model (REML with Hartung-Knapp adjustment); 5 studies; 1563 participants; I2 = 0.0%

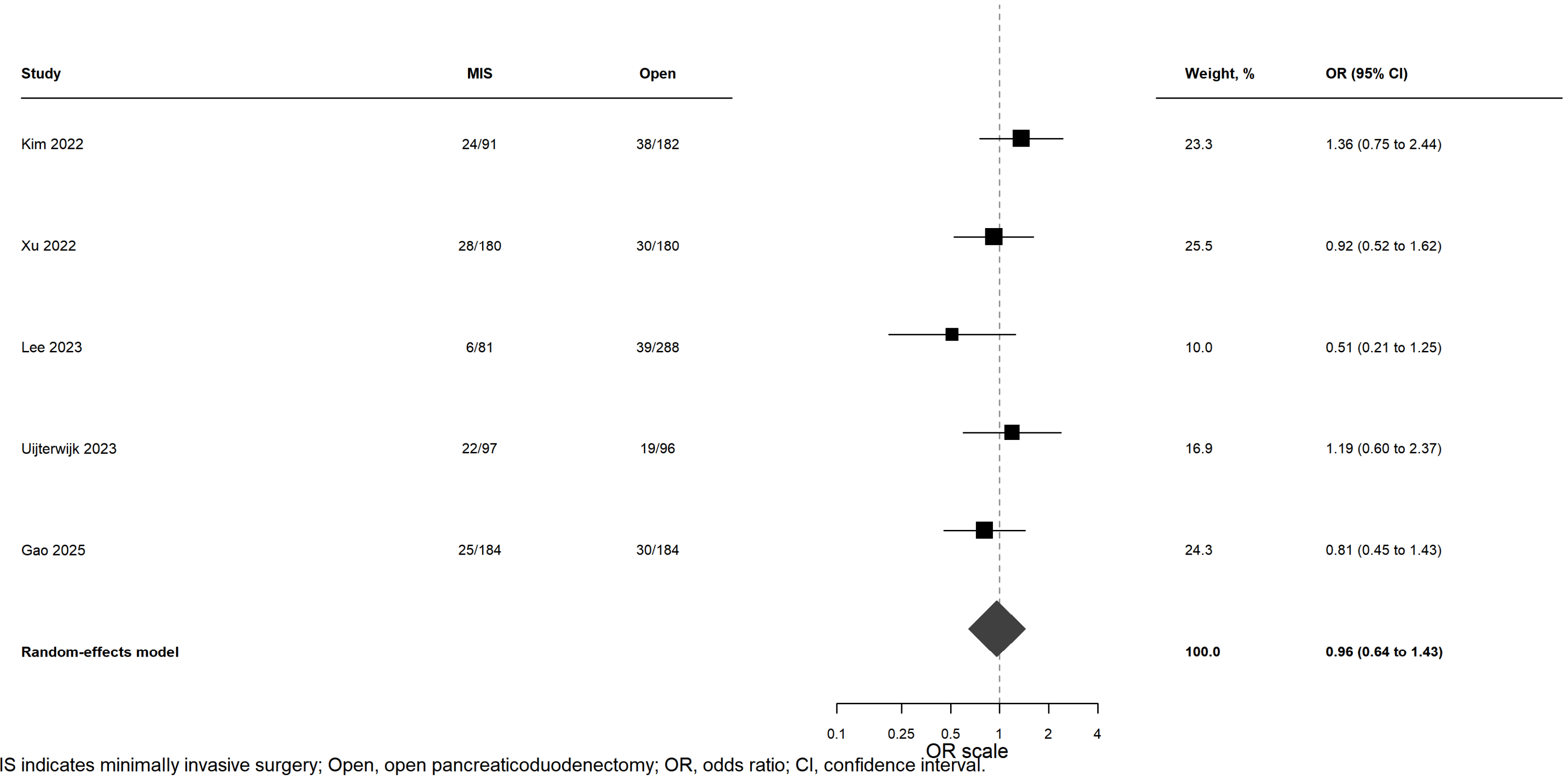

MIS indicates minimally invasive surgery; Open, open pancreaticoduodenectomy; OR, odds ratio; CI, confidence interval.

Figure S5. Delayed gastric emptying forest plot.

Random-effects model (REML with Hartung-Knapp adjustment); 4 studies; 981 participants; I2 = 0.0%

Study	MIS	Open	Weight, %	OR (95% CI)
Zhu 2022	2/30	4/30	4.0	0.46 (0.08 to 2.75)
Xu 2022	22/180	21/180	31.2	1.05 (0.56 to 1.99)
Uijterwijk 2023	25/97	30/96	32.2	0.76 (0.41 to 1.43)
Gao 2025	22/184	23/184	32.6	0.95 (0.51 to 1.77)
**Random-effects model**			**100.0**	**0.89 (0.63 to 1.25)**

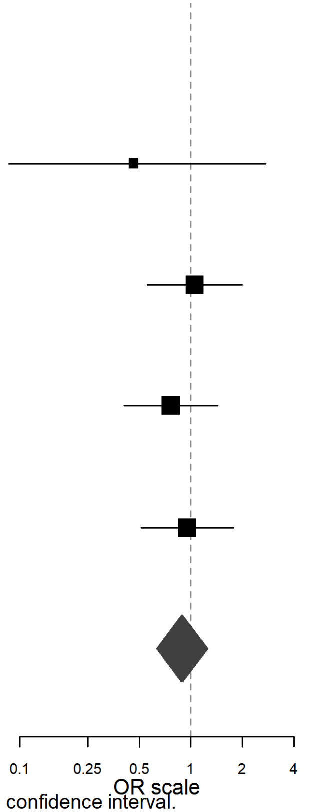

MIS indicates minimally invasive surgery; Open, open pancreaticoduodenectomy; OR, odds ratio; CI, confidence interval.

Figure S6. Early mortality forest plot.

Random-effects model (REML with Hartung-Knapp adjustment); 4 studies; 1194 participants; I2 = 0.0%

Study	MIS	Open	Weight, %	OR (95% CI)
Kim 2022	0/91	3/182	6.5	0.28 (0.01 to 5.48)
Xu 2022	5/180	6/180	43.3	0.84 (0.27 to 2.67)
Uijterwijk 2023	3/97	2/96	21.3	1.40 (0.27 to 7.27)
Gao 2025	3/184	4/184	28.9	0.77 (0.19 to 3.17)
**Random-effects model**			**100.0**	**0.85 (0.43 to 1.68)**

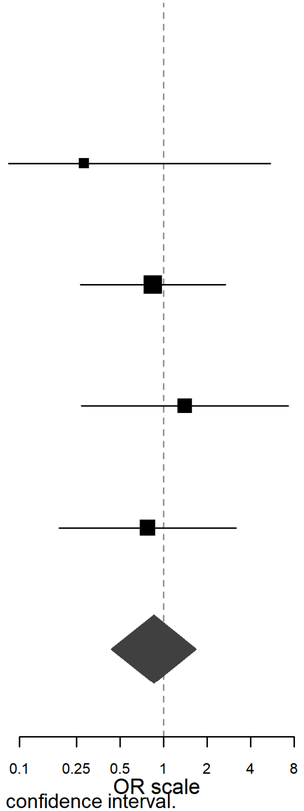

MIS indicates minimally invasive surgery; Open, open pancreaticoduodenectomy; OR, odds ratio; CI, confidence interval.

Figure S7. Operative time forest plot.

Random-effects model (REML with Hartung-Knapp adjustment); 5 studies; 1563 participants; I2 = 95.7%

Study	MIS mean (SD)	Open mean (SD)	Weight, %	MD (95% CI)
Kim 2022	457.0 (70.0)	398.0 (85.0)	20.3	59.0 (40.0 to 78.0)
Xu 2022	269.2 (46.3)	263.0 (45.9)	20.9	6.2 (-3.4 to 15.7)
Lee 2023	366.2 (92.8)	279.1 (66.9)	20.1	87.1 (65.5 to 108.7)
Uijterwijk 2023	459.7 (130.4)	340.0 (115.6)	18.7	119.7 (84.9 to 154.4)
Gao 2025	303.3 (103.7)	304.0 (105.9)	20.1	-0.7 (-22.1 to 20.8)
**Random-effects model**			**100.0**	**53.0 (-10.8 to 116.7)**

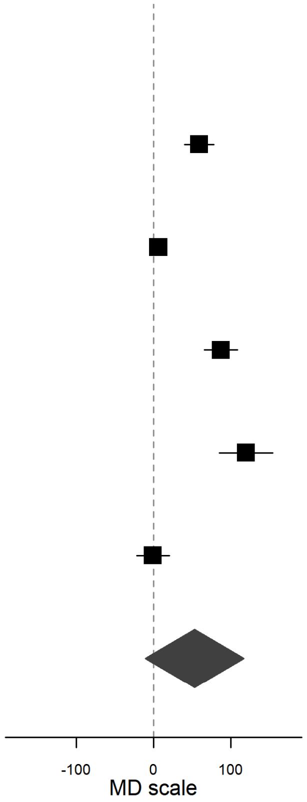

MD indicates mean difference; CI, confidence interval.

Figure S8. Postoperative length of stay forest plot.

Random-effects model (REML with Hartung-Knapp adjustment); 5 studies; 1563 participants; I2 = 82.6%

Study	MIS mean (SD)	Open mean (SD)	Weight, %	MD (95% CI)
Kim 2022	19.8 (11.3)	26.6 (14.3)	15.7	-6.80 (-9.92 to -3.68)
Xu 2022	13.0 (5.2)	15.0 (4.4)	23.9	-2.00 (-3.00 to -1.00)
Lee 2023	12.0 (4.0)	12.0 (3.0)	24.1	0.00 (-0.94 to 0.94)
Uijterwijk 2023	18.3 (13.3)	20.3 (15.6)	12.3	-2.00 (-6.09 to 2.09)
Gao 2025	13.3 (5.2)	15.3 (4.4)	24.0	-2.00 (-2.99 to -1.01)
**Random-effects model**			**100.0**	**-2.27 (-5.22 to 0.68)**

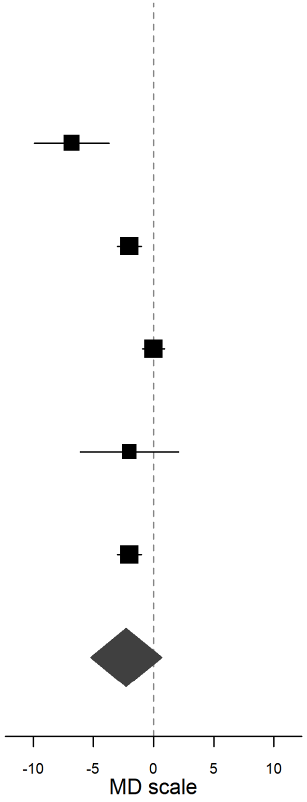

MD indicates mean difference; CI, confidence interval.

Figure S9. Alternative R0 sensitivity model including the 1-mm margin-rule cohort.

Alternative sensitivity model; random-effects model (REML with Hartung-Knapp adjustment); 6 studies; 1623 participants; I2 = 0.0%

Study	MIS	Open	Weight, %	OR (95% CI)
Zhu 2022	28/30	28/30	2.3	1.00 (0.13 to 7.60)
Kim 2022	87/91	169/182	7.3	1.67 (0.53 to 5.28)
Xu 2022	161/180	154/180	24.2	1.43 (0.76 to 2.69)
Lee 2023	67/81	233/288	23.1	1.13 (0.59 to 2.16)
Gao 2025	167/184	167/184	19.4	1.00 (0.49 to 2.03)
Uijterwijk 2023 [derived from R1 <1 mm]	74/97	67/96	23.6	1.39 (0.73 to 2.64)
**Random-effects model**			**100.0**	**1.26 (1.04 to 1.52)**

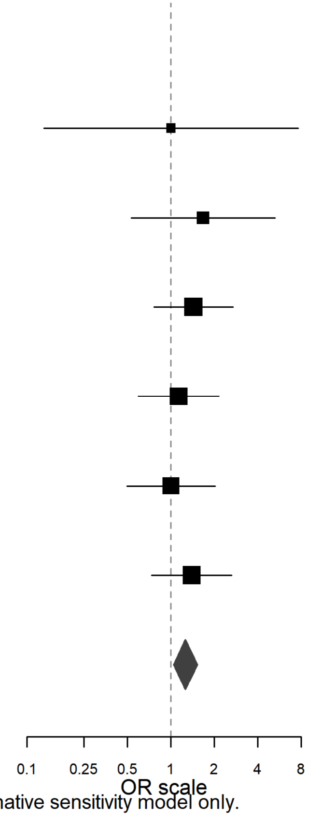

The Uijterwijk 2023 row was derived from reported R1 (<1 mm) counts and is displayed as an alternative sensitivity model only.

Figure S10. Lymph-node yield forest plot.

Random-effects model (REML with Hartung-Knapp adjustment); 5 studies; 1563 participants; I2 = 88.2%

Study	MIS mean (SD)	Open mean (SD)	Weight, %	MD (95% CI)
Kim 2022	14.9 (7.8)	20.7 (11.5)	18.5	-5.80 (-8.12 to -3.48)
Xu 2022	13.7 (4.4)	12.7 (3.7)	21.6	1.00 (0.15 to 1.85)
Lee 2023	17.2 (6.7)	18.7 (7.7)	20.0	-1.50 (-3.21 to 0.21)
Uijterwijk 2023	16.0 (8.9)	14.7 (5.9)	19.0	1.33 (-0.80 to 3.46)
Gao 2025	13.7 (6.7)	13.7 (5.9)	20.9	0.00 (-1.29 to 1.29)
**Random-effects model**			**100.0**	**-0.90 (-4.41 to 2.61)**

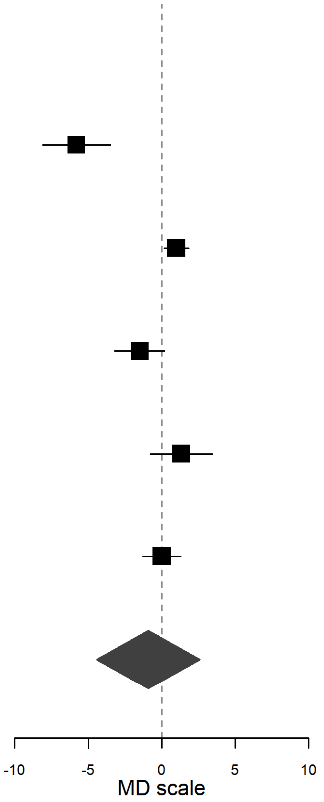

MD indicates mean difference; CI, confidence interval.

Figure S11. Direct plus reconstructed overall-survival sensitivity model.

Pre-planned sensitivity analysis; random-effects model (REML with Hartung-Knapp adjustment); 5 studies; 1563 participants; I2 = 0.0%

Study	Source	Participants	Weight, %	HR (95% CI)
Kim 2022 [Direct HR]	Direct HR	273	12.8	0.89 (0.57 to 1.37)
Xu 2022 [Direct HR]	Direct HR	360	32.4	1.02 (0.78 to 1.35)
Lee 2023 [Direct HR]	Direct HR	369	5.1	0.60 (0.30 to 1.20)
Gao 2025 [Reconstructed HR]	Reconstructed HR	368	35.5	0.81 (0.62 to 1.05)
Uijterwijk 2023 [Reconstructed HR]	Reconstructed HR	193	14.2	0.87 (0.57 to 1.31)
**Random-effects model**			**100.0**	**0.88 (0.73 to 1.05)**

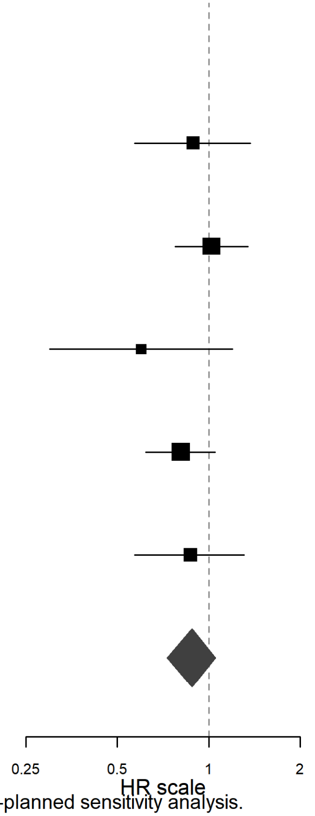

HR indicates hazard ratio; CI, confidence interval. Zhu 2022 was reviewed but not pooled in this pre-planned sensitivity analysis.
